# The transcriptional program underlying the physiology of clostridial sporulation

**DOI:** 10.1186/gb-2008-9-7-r114

**Published:** 2008-07-16

**Authors:** Shawn W Jones, Carlos J Paredes, Bryan Tracy, Nathan Cheng, Ryan Sillers, Ryan S Senger, Eleftherios T Papoutsakis

**Affiliations:** 1 Department of Chemical and Biological Engineering, Northwestern University, Sheridan Road, Evanston, IL 60208-3120, USA; 2 Department of Chemical Engineering, University of Delaware, Academy Street, Newark, DE 19716, USA; 3 Delaware Biotechnology Institute, University of Delaware, Innovation Way, Newark, DE 19711, USA; 4 Current address: Cobalt Biofuels, Clyde Avenue, Mountain View, CA 94043, USA; 5 Current address: The Zitter Group, New Montgomery Street, San Francisco, CA 94105, USA

## Abstract

**Background:**

Clostridia are ancient soil organisms of major importance to human and animal health and physiology, cellulose degradation, and the production of biofuels from renewable resources. Elucidation of their sporulation program is critical for understanding important clostridial programs pertaining to their physiology and their industrial or environmental applications.

**Results:**

Using a sensitive DNA-microarray platform and 25 sampling timepoints, we reveal the genome-scale transcriptional basis of the *Clostridium acetobutylicum *sporulation program carried deep into stationary phase. A significant fraction of the genes displayed temporal expression in six distinct clusters of expression, which were analyzed with assistance from ontological classifications in order to illuminate all known physiological observations and differentiation stages of this industrial organism. The dynamic orchestration of all known sporulation sigma factors was investigated, whereby in addition to their transcriptional profiles, both in terms of intensity and differential expression, their activity was assessed by the average transcriptional patterns of putative canonical genes of their regulon. All sigma factors of unknown function were investigated by combining transcriptional data with predicted promoter binding motifs and antisense-RNA downregulation to provide a preliminary assessment of their roles in sporulation. Downregulation of two of these sigma factors, CAC1766 and CAP0167, affected the developmental process of sporulation and are apparently novel sporulation-related sigma factors.

**Conclusion:**

This is the first detailed roadmap of clostridial sporulation, the most detailed transcriptional study ever reported for a strict anaerobe and endospore former, and the first reported holistic effort to illuminate cellular physiology and differentiation of a lesser known organism.

## Abstract 2

A detailed microarray analysis of transcription during sporulation of the strict anaerobe and endospore former *Clostridium acetobutylicum* is presented.

## Background

Clostridia are of major importance to human and animal health and physiology, cellulose degradation, bioremediation, and for the production of biofuels and chemicals from renewable resources [1]. These obligate anaerobic, Gram-positive, endospore-forming firmicutes include several major human and animal pathogens, such as *C. botulinum*, *C. perfringens*, *C. difficile*, and *C. tetani*, the cellulolytic *C. thermocellum *and *C. phytofermentans*, several ethanologenic [2], and many solventogenic (butanol, acetone and ethanol) species [3]. Their sporulation/differentiation program is critical for understanding important cellular functions or programs, yet it remains largely unknown. We have recently examined the similarity of the clostridia and bacilli sporulation programs using information from sequenced clostridial genomes [1]. We concluded that, based on genomic information alone, the two programs are substantially different, reflecting the different evolutionary age and roles of these two genera. We have also argued that *C. acetobutylicum *is a good model organism for all clostridia [1]. Transcriptional or functional genomic information is, however, necessary for detailing these differences and for understanding clostridial differentiation and physiology. Key issues awaiting resolution include: the identification of the mid to late sigma and sporulation factors and their regulons; the orchestration and timing of their action; the set of genes employed by the cells in the mid and late stages of spore maturation; identification of candidate histidine kinases that might be capable of phosphorylating the master regulator (Spo0A) of sporulation; and some functional assessment of the roles of several sigma factors of unknown function encoded by the *C. acetobutylicum *genome. Furthermore, an understanding of the transcriptional basis of the complex physiology of this organism will go a long way to improve our ability to metabolically engineer, for practical applications, its complex sporulation and metabolic programs. Such information generates tremendous new opportunities for further exploration of this complex anaerobe and its clostridial relatives, and constitutes a firm basis for future detailed genetic and functional studies.

Using a limited in scope and resolution transcriptional study, we have previously shown that it is possible to use DNA-microarray-based transcriptional analysis to generate valuable functional information related to stress response [4,5], initiation of sporulation [6] and the early sporulation program of *C. acetobutylicum *[7]. In order to be able to accurately study the transcriptional orchestration underlying the complete sporulation program of the cells, it was necessary to develop a more sensitive and accurate microarray platform, a better mRNA isolation protocol (in order to isolate RNA from the mid and late stationary phases), as well as to use a much higher frequency of observation and sampling. We also aimed to employ more sophisticated bioinformatic tools in order to globally interrogate any desirable cellular program and relate it to the characteristic phenotypic metabolism and sporulation of this organism. The results of this extensive study are presented here as a single, undivided story, which offers unprecedented insights and a tremendous wealth of information for further explorations. Furthermore, it serves as a paradigm of what can be effectively accomplished with the now highly accurate DNA-microarray analysis in generating a robust transcriptional roadmap and in illuminating the physiology of a lesser understood organism.

## Results and discussion

### Metabolism and differentiation of *C. acetobutylicum*: identification of a new cell type?

**Figure 1:**
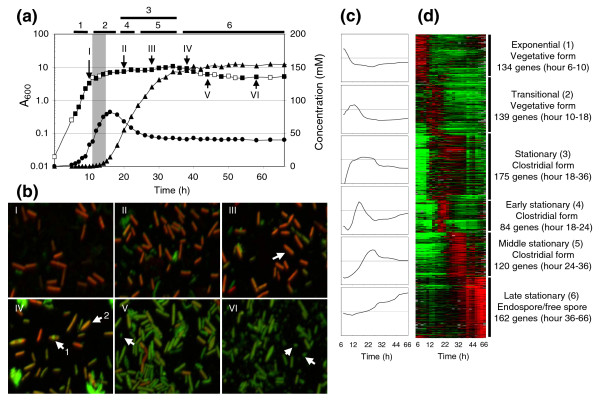
Morphological and gene expression changes *C. acetobutylicum *undergoes during exponential, transitional, and stationary phases. **(a) **Growth and acid and solvent production curves as they relate to morphological and transcriptional changes during sporulation. The gray bar indicates the beginning of the transitional phase as determined by solvent production. A_600 _with microarray sample (filled squares); A_600 _(open squares); butyrate (filled circles); butanol (filled triangles). Roman numerals correspond with those in (b), and bars and numbers along the top correspond to the clusters in (c). **(b) **Morphological changes during sporulation. When stained with Syto-9 (green) and PI (red), vegetative cells take on a predominantly red color (I and II). At peak butanol production, swollen, cigar-shaped clostridial-form cells appear (arrow in III), which stain almost equally with both dyes, and persist until late stationary phase. Towards the end of solvent production (IV), endospore (arrow 1) forms are visible, and clostridial (arrow 2) forms are still present. As the culture enters late stationary phase (V and VI), cells stain almost exclusively green, regardless of morphology. All cell types are still present, including free spores (arrows in V and VI), and vegetative cells identified by their motility. **(c) **Average expression profiles for each K-means cluster generated using a moving average trendline with period 3. **(d) **Expression of the 814 genes (rows) at 25 timepoints (columns, hours 6, 7, 8, 9, 10, 12, 14, 16, 18, 20, 22, 24, 26, 28, 30, 32, 34, 36, 38, 40, 44, 48, 54, 58, and 66). Genes with higher expression than the reference RNA are shown in red and those with lower expression as green. Saturated expression levels: ten-fold difference.

We aimed to relate the metabolic and morphological characteristics of the cells in a typical batch culture, whereby cells underwent a full differentiation program, to the transcriptional profile of the cell population [8]. The metabolism of solventogenic clostridia is characterized by an initial acidogenic phase followed by acid re-assimilation and solvent production [7]. As shown in Figure 1a, the peak of butyrate concentration, around 16 hours after the start of the culture, coincided with the initiation of butanol production. Around this time, the culture transitioned from exponential growth to stationary phase and initiated solventogenesis and sporulation. This period is called the transitional phase and is indicated by the gray bar in Figure 1a and all following figures. The butanol concentration increased to over 150 mM until hour 45, after which no substantial change in solvent or acid concentration took place. Nevertheless, cells continued to display morphological changes well past hour 60. Solventogenic clostridia display a series of morphological forms over this differentiation program: vegetative, clostridial, forespore, endospore, and free-spore forms [9]. In addition to phase-contrast microscopy, we found that by using Syto-9 (a green dye assumed to stain live cells) and propidium iodide (PI; a red dye assumed to stain dead cells) [10] we could microscopically distinguish these morphologies and identify new cell subtypes. Staining by these two dyes did not follow typical expectations. During exponential growth, vegetative cells, characterized by a thin-rod morphology, were visibly motile under the microscope, which is consistent with the finding that chemotaxis and motility genes were highly expressed during this time [7]. When double stained with Syto-9 and PI dyes, these vegetative cells took on a predominantly red color, indicating the uptake of more PI than Syto-9 (Figure 1b, I, II). At the onset of butanol production, swollen, cigar-shaped clostridial-form cells began to appear (Figure 1b, III). These clostridial forms (confirmed by phase-contrast microscopy; data not shown), generally assumed to be the cells that produce solvents [8], were far less motile than exponential-phase cells and stained almost equally with both dyes, taking on an orange color. Clostridial forms persisted until solvent production decreased, after which forespore forms (cells with one end swollen, which is indicative of a spore forming) and endospore forms (cells with the middle swollen, which is indicative of a developing spore) became visible [9]. These cells stained almost exclusively green, indicating an uptake of more Syto-9 than PI (Figure 1b, IV-VI). The sporulation process is completed when the mother cell undergoes autolysis to release the mature spore. Mature free spores could be seen as early as hour 44 (Figure 1b, V). Later, around hour 58 (Figure 1b, VI), a portion of the cells became motile again. Though these cells appear like vegetative cells, they stained predominantly green, instead of red, and did not produce appreciable amounts of acid. We hypothesize that this staining change reflects modifications in membrane composition due to different environmental conditions (presence of solvents and other metabolites) rather than cell viability and assume that this newly identified cell type has different transcriptional characteristics, which we tested next.

### The transcriptional program of clostridial differentiation

**Figure 2:**
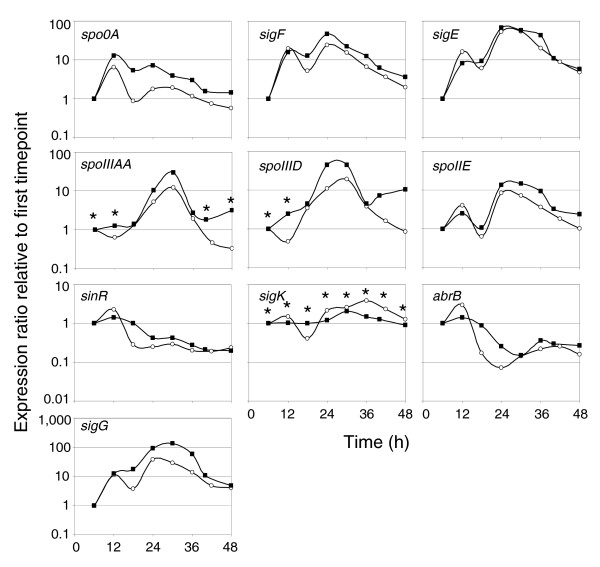
Q-RT-PCR and microarray data comparison. RNA from a biological replicate bioreactor experiment was reverse transcribed into cDNA for the Q-RT-PCR. All expression ratios are shown relative to the first timepoint for both Q-RT-PCR (open circles) and microarray data (filled squares). Asterisks represent data below the cutoff value for microarray analysis. Samples were taken every six hours starting from hour 6 and continuing until hour 48. The genes examined were from several operons with different patterns of expression.

To ensure that important transcriptional, physiological, and morphological changes were captured [7,8], RNA samples were taken every hour during exponential phase and every two hours after that until late stationary phase when sampling frequency decreased. mRNA from 25 timepoints (Figure 1a) were selected for transcriptional analysis by hybridizing pairs of 22k oligonucleotide microarrays on a dye swap configuration using an mRNA pool as reference. There were 814 genes, or 21% of the genome, that surpassed the threshold of expression in at least 20 of the 25 microarray timepoints and had two or more timepoints differentially expressed at a 95% confidence level [11]; these genes were classified as having a temporal differential expression profile. We chose these strict selection criteria in order to robustly identify the key expression patterns of the differentiation process. We relaxed these criteria in subsequent gene ontology-driven analyses. Expression data were extensively validated by, first, quantitative reverse transcription PCR (Q-RT-PCR) analysis (focusing on key sporulation factors) from a biological replicate culture (Figure 2), and, second, by systematic comparison to our published (but limited in scope and duration) microarray study (see Additional data file 1 for Figure S1 and discussion).

Six distinct clusters of temporal expression patterns were selected (Figure 1c,d) by K-means to achieve a balance between inter- and intra-cluster variability. To examine transcriptional changes in larger functional groups (for example, transcription, motility, translation), each cluster was analyzed according to the Cluster of Orthologous Groups of proteins (COG) classification [12] and the functional genome annotation [13]. To determine if a COG functional group was overrepresented in any of the K-means clusters, first the percentage of each group in the genome was determined, and then the percentage of each group was determined in each of the K-means clusters. By comparing the percentage in the K-means clusters to the genome percentage, we could identify overrepresented groups (Additional data file 2).

#### Exponential phase: motility, chemotaxis, nucleotide and primary metabolism

The first cluster contains 134 genes highly expressed during exponential growth (hours 6 to 10; see Additional data file 2 for a list of the genes). This cluster characterizes highly motile vegetative cells (Figure 1b, I) and, given the minimal amount of knowledge on the genes responsible for motility and chemotaxis in clostridia, our analysis offers the possibility of identifying these genes at the genome scale [14]. This cluster includes the flagella structural components flagellin and *flbD*, the main chemotaxis response regulator, *cheY *(CAC0122; responsible for flagellar rotation in *B. subtilis* [15]), as well as several methyl-accepting chemotaxis receptor genes (CAC0432, CAC0443, CAC0542, CAC1600, CAP0048). COG analysis showed that genes related to cell motility (COG class N) and nucleotide transport and metabolism (COG class F) were overrepresented in this cluster (Additional data file 2). In order to investigate cell motility further, all genes that fell within this COG class were hierarchically clustered according to their expression profiles (see Additional data file 3 for Figure S2 and discussion). Interestingly, the two main cell motility gene clusters, the first including most of the flagellar assembly and motor proteins and the second containing most of the known chemotaxis proteins, clustered together and displayed a bimodal expression pattern (Figure S2). The genes were not only expressed during exponential phase but also during late stationary phase, around hour 38, which is consistent with the observation that a motile cell population was again observed in late stationary phase. Included in the category of nucleotide transport and metabolism are several purine and pyrimidine biosynthesis genes: a set of five consecutive genes, *purECFMN*, the bi-functional *purQ/L *gene, *purA*, *pyrPR*, *pyrD*, and *pyrI*. Two other purine synthesis genes (*purH*, *purD*) showed very similar profiles but were not classified within this cluster by the clustering algorithm. Vegetative cells, which correspond to this cluster, produce ATP through acidogenesis, whereby the cells uptake glucose and convert it to acetic and butyric acid. Because glucose is the main energy source, multiple genes for glucose transport were included within this cluster, including the glucose-specific phosphotransferase gene, *ptsG*, the glucose kinase *glcK *and CAP0131, the gene most similar to *B. subtilis *glucose permease *glcP*. The genes required for the metabolism of glucose to pyruvate did not show temporal regulation, suggesting that expression of these genes is constitutive-like (see Additional data file 3 for Figure S3 and discussion). Acetic acid production genes *pta *and *ack *were not temporally expressed, but butyrate production genes *ptb *and *buk *were. Though expressed throughout exponential phase, the expression of both *ptb *and *buk *slightly peaked during late exponential phase, as previously seen [7], and thus fall in the transitional (second) cluster. Analysis of the expression patterns of all the genes involved in acidogenesis, not just the differentially expressed genes discussed here, is included in Figure S3 in Additional data file 3. Finally, the expression patterns of the two classes of hydrogenases (iron only and nickel-iron) were investigated (Figure S3 in Additional data file 3). *hydA*, the iron only hydrogenase that catalyzes the production of molecular hydrogen, was expressed only during exponential phase, whereas the iron-nickel hydrogenase, *mbhS *and *mbhL*, was expressed throughout stationary phase.

#### Initiation of sporulation: abrB, sinR, lipid and iron metabolism

The transitional phase is captured by 139 genes in the second cluster (Figure 1c,d; Additional data file 2). It is made up of genes that show elevated expression between hours 10 and 18 and is when solvent formation was initiated. This cluster characterizes the shift from vegetative cells to cells committing to sporulation and thus includes two important regulators of sporulation, *abrB *(CAC0310) and *sinR *(CAC0549), which are discussed in more detail below. Also characteristic of this shift from vegetative growth to sporulation was the overrepresentation of genes related to energy production and conversion (COG class C), since sporulation is an energy intensive process. Solvent production began in the transitional phase, though the genes responsible for solvent production fall in the next (third) cluster; the third cluster partially overlaps with this second cluster but is distinguished by a sustained expression pattern. In response to these solvents, *C. acetobutylicum *undergoes a change in its membrane composition and fluidity, generally decreasing the ratio between unsaturated to saturated fatty acids [16-18]. Consistent with this change, genes related to lipid metabolism (COG class I) were overrepresented in this cluster. To further investigate this COG class, all genes identified as COG class I were hierarchically clustered (see Additional data file 3 for Figure S4 and discussion). Seven genes that were upregulated just before the onset of sporulation fall within the same operon and are related to fatty acid synthesis. In contrast, many of the most characterized genes involved in fatty acid synthesis (*accBC*, *fabDFZ*, and *acp*) maintain a fairly flat profile throughout the timecourse (Figure S4 in Additional data file 3). Also within this cluster is the gene responsible for cyclopropane fatty acid synthesis (*cfa*), though classified in COG class M (cell envelope biogenesis) and not COG class I. Importantly, the ratio of cyclopropane fatty acids in the outer membrane has been shown to increase as cells enter stationary phase [18,19], but the overexpression of this gene alone was unable to produce a solvent tolerant strain [19]. Though not overrepresented in this cluster, all the genes within COG class M were also hierarchically clustered (see Additional data file 3 for Figure S5 and discussion). The transitional cluster also included several genes related to iron transport and regulation like the *fur *family iron uptake regulator CAC2634, the iron permease CAC0788, *feoA*, *feoB*, *fhuC*, and two iron-regulated transporters (CAC3288, CAC3290), which is consistent with the earlier, more limited data [7]. Significantly, iron-limitation has been found to promote solventogenesis [20].

#### Solventogenesis, clostridial form, stress proteins, and early sigma factors

The third cluster (Figure 1c,d; Additional data file 2) of 175 upregulated genes represents the solventogenic/stationary phase as it contains all key solventogenic genes. This cluster characterizes the transcriptional pattern of clostridial cells, the unique developmental stage in clostridia and first recognizable cell type of the sporulation cascade, and exhibited a longer upregulation of gene expression than the previous two clusters. Indeed, its range overlapped the previous (second) and the next two (fourth and fifth) clusters. The clostridial form is generally recognized to be the form responsible for solvent production [8,21] and is distinguished morphologically as swollen cell forms with phase bright granulose within the cell [21]. This cluster captures both of these characteristics with the inclusion of the solventogenic genes and several granulose formation genes. The solventogenic genes *adhE1*-*ctfA*-*ctfB*, *adc*, and *bdhB *were initially induced during transitional phase, the second cluster, but were expressed throughout stationary phase and were thus placed within this cluster. Two granulose formation genes, *glgC *(CAC2237) and CAC2240, and a granulose degradation gene, *glgP *(CAC1664), were included within this cluster. The other two granulose formation genes, *glgD *(CAC2238) and *glgA *(CAC2239), though not included in this cluster, displayed a similar expression profile to *glgC *and CAC2240. The concomitant requirement of NADH during butanol production drove the expression of three genes involved in NAD formation: *nadABC*. Expression of the stress-response gene *hsp18*, a heat-shock related chaperone, and the *ctsR*-*yacH*-*yacI*-*clpC *operon, containing the molecular chaperone *clpC *and the stress-gene repressor *ctsR*, also fell in this cluster and paralleled the expression of the solventogenic genes (see Additional data file 3 for Figure S6). Other important stress-response genes, *groEL*-*groES *(CAC2703-04) and *hrcA*-*grpE*-*dnaK*-*dnaJ *(CAC1280-83), mirrored this expression pattern, though were not differentially expressed according to the strict criteria employed for selecting the genes of Figure 2c,d (Figure S6 in Additional data file 3). Although genes encoded on the pSOL1 megaplasmid [22] represent less than 5% of the genome, they constitute 15% of genes in this cluster. pSOL1 harbors all essential solvent-formation genes and, importantly, some unknown gene(s) essential for sporulation [22]. Besides the genes listed in this cluster, the vast majority of the genes located on pSOL1 were expressed throughout stationary phase, with most being upregulated at the onset of solventogenesis (see Additional data file 3 for Figure S7). Several key sporulation-specific sigma factors (σ^F^, σ^E^, σ^G^) and the σ^F^-associated anti-sigma factors in the form of the tricistronic *spoIIA *operon (CAC2308-06) belong to this cluster along with one of the two paralogs of *spoVS *(CAC1750) and one of three *spoVD *paralogs (CAP0150). The second *spoVS *paralog (CAC1817) did not meet the threshold of expression in 12 of the 25 timepoints; the other two paralogs of *spoVD *(CAC0329, CAC2130) were above the expression cutoff but did not show significant temporal regulation. Of unknown significance was the expression of a large cluster of genes involved in the biosynthesis of the branched-chain amino acids valine, leucine and isoleucine (CAC3169-74) coinciding with the onset of solventogenesis, as shown before [7,23], as well as the upregulation of several glycosyltranferases (see Additional data file 3 for Figure S8). The upregulation of valine, leucine, and isoleucine synthesis genes could be indicative of a membrane fluidity adaptation [7]. In *B. subtilis*, these branched-chain amino acids can be converted into branched-chain fatty acids and change the membrane fluidity [24], and under cold shock stress, *B. subtilis *downregulates a number of genes related to valine, leucine, and isoleucine synthesis [25]. Therefore, this upregulation may be another mechanism to change membrane fluidity, though the ratio of unbranched and branched fatty acids has not been reported in studies investigating membrane composition [16-18,26].

#### Stationary phase carbohydrate (beyond glucose) and amino acid metabolism

The fourth cluster (Figure 1c,d; Additional data file 2) of 84 genes represents a sharp induction of expression between 18 and 24 hours (early stationary phase). This cluster falls within the stationary (third) cluster described above. This is a compact group, with 70% belonging to one of three COG categories: carbohydrate transport and metabolism, transport and metabolism of amino acids, and inorganic ion transport and metabolism. A number of different carbohydrate substrate pathways, from monosaccharides (fructose, galactose, mannose, and xylose) to disaccharides (lactose, maltose, and sucrose) to complex carbohydrates (cellulose, glycogen, starch, and xylan), were investigated, and many exhibited upregulation during stationary phase, though only a few are highly expressed (see Additional data file 3 for Figure S9). The significance of this upregulation of non-glucose pathways is unknown, because sufficient glucose remains in the media (approximately 200 mM or about 44% of the initial glucose level). Of particular interest was the upregulation of several genes related to starch and xylan degradation (Figure S9 in Additional data file 3). The two annotated α-amylases (CAP0098 and CAP0168) along with the less characterized glucosidases and glucoamylase were all upregulated throughout stationary phase and a number were highly expressed, like CAC2810 and CAP0098. Also upregulated were the predicted xylanases CAC2383, CAP0054, and CAC1037, with CAP0054 and CAC1037 being highly expressed during stationary phase. Mirroring this pattern were CAC1086, a xylose associated transcriptional regulator, and the highly expressed CAC2612, a xylulose kinase. The genes related to glycogen metabolism are believed to be involved in granulose formation, as discussed earlier. Several genes for arginine biosynthesis (*argF*, *argGH*, *argDB*, *argCJ*, *carB*) were induced during this time, probably as a result of its depletion in the culture medium.

#### Genes underlying the activation of the sporulation machinery and the genes for tryptophan and histidine biosynthesis

The fifth cluster (Figure 1c,d; Additional data file 2), representing the middle stationary phase, contains 120 genes mainly expressed between hours 24 and 36, and again falls within the stationary (third) cluster described above. Most of the genes in this cluster activate the sporulation-related sigma factors (σ^F^, σ^E^, σ^G^) or are putatively regulated by them. These include *spoIIE*, the phosphatase that dephosphorylates SpoIIAA and results in the activation of σ^F^, and the σ^E^-dependent operons *spoVR *(involved in cortex synthesis), *spoIIIAA*-*AH *(required for the activation of σ^G^), and *spoIVA *(involved in cortex formation and spore coat assembly). The σ^G^-dependent *spoVT *gene has two paralogs in *C. acetobutylicum *(CAC3214, CAC3649); the transcriptional pattern suggests that CAC3214, included in this cluster, is the real *spoVT*. Sporulation-related genes included in this cluster are three *cotF *genes, one *cotJ *gene, one *cotS *gene, the spore maturation protein B, a small acid soluble protein (CAC2365), and two spore lytic enzymes (CAC0686, CAC3244). Though several sporulation-related genes are included in the next (sixth) cluster as well, most, beyond those listed here, are upregulated in mid-stationary phase (see Additional data file 3 for Figure S10 and discussion). Seven genes of the putative operon (CAC3157-63) encoding genes for tryptophan synthesis from chorismate and ten genes for histidine synthesis (CAC0935-43, CAC3031) were also included here.

#### Spore maturation and late-stationary phase vegetative cells

The sixth cluster, representative of the late stationary phase, includes 162 genes mainly expressed after hour 36 (Figure 1c,d; Additional data file 2). This cluster captured the expression profiles of the forespore and endospore forms, free spores, and late-stage vegetative-like cells. The endospore form represents the last stage before mature spores are released, and therefore fewer sporulation-related genes are within this cluster than previous ones. The sporulation-related genes included in this cluster are two small acid-soluble proteins (CAC1522 and CAC2372), a spore germination protein (CAC3302), a spore coat biosynthesis protein (CAC2190) and a spore protease (CAC1275). Also within this cluster are the two phosphotransferase genes, CAC2958 (a galactitol-specific transporter) and CAC2965 (a lactose-specific transporter), another annotated *cheY *(CAC2218), various enzymes related to different sugar pathways (CAC2180, CAC2250, CAC2954), and two glycosyltransferases (CAC2172, CAC3049). Expression of these genes may be reflective of the late-stage vegetative-like cells observed during microscopy and demonstrate they have a different genetic profile compared to the early vegetative cells. Interestingly, this cluster is enriched in defense mechanism genes (COG class V) like a phospholipase (CAC3026) and multidrug transporters that may play a role in resistance to a variety of environmental toxins.

#### General processes: cell division and ribosomal proteins

Two additional gene classes (cell division and ribosomal proteins), though not overrepresented in any of the six clusters described above, were investigated because of their importance in cellular processes and interesting expression patterns. COG class D (cell division and chromosome partitioning), besides important genes for vegetative symmetric division, includes *ftsAZ*, important for both symmetric and asymmetric cell division, and *soj *(a regulator of *spo0J*) and *spoIIIE*, important for proper chromosomal partitioning between the mother cell and prespore. These genes, along with several uncharacterized genes, were upregulated at the beginning of sporulation (see Additional data file 3 for Figure S11). Almost all the ribosomal proteins were downregulated as the culture entered stationary phase, and interestingly, about half of those downregulated genes were again upregulated in mid-stationary phase and remained upregulated until late-stationary phase (see Additional data file 3 for Figure S12). This upregulation is likely related to the late-stage vegetative-like cells seen.

### Expression and activity patterns of sporulation-related sigma factors and related genes

#### Expression of sporulation transcription factors

**Figure 3:**
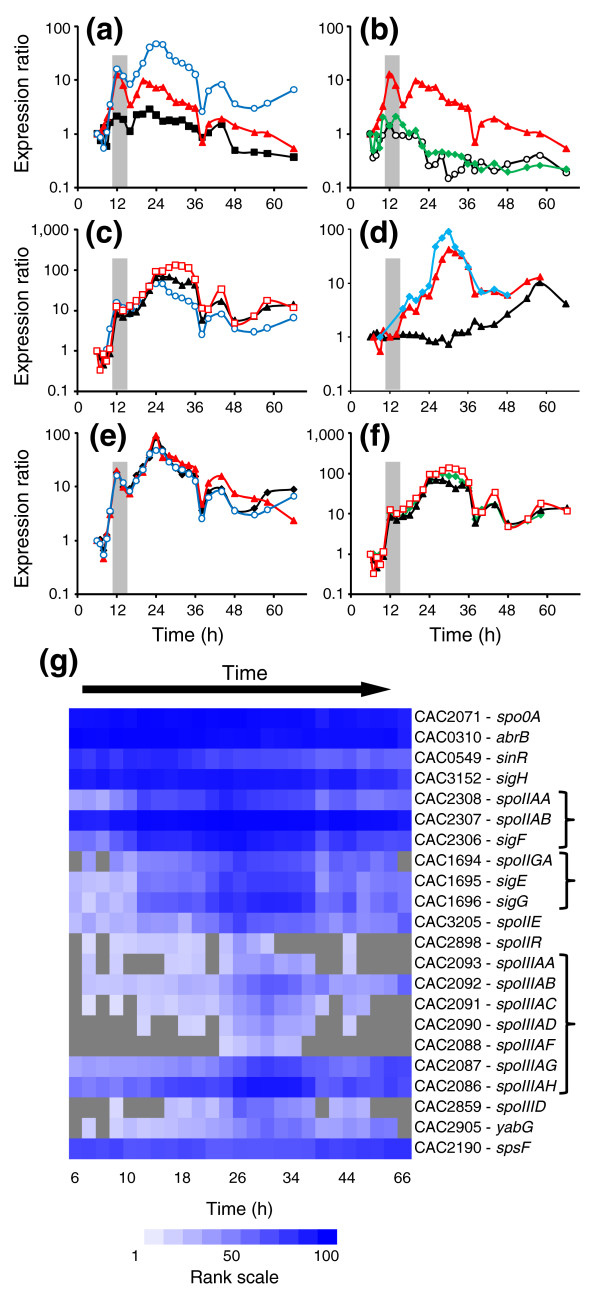
Investigation of the sporulation cascade in *C. acetobutylicum*. **(a-f) **Expression profiles of sporulation genes shown as ratios against the first expressed timepoint. **(a) **The first three sporulation factors: *spo0A *(red filled triangles), *sigH *(black filled squares), and *sigF *(open blue circles). **(b) ***spo0A *(red filled triangles) and possible sporulation regulators: *abrB *(open black circles) and *sinR *(green filled diamonds). **(c) **Sporulation factors downstream of *spo0A*: *sigF *(open blue circles), *sigE *(black filled triangles), and *sigG *(open red squares). **(d) **Genes related to *sigK *expression: *spoIIID *(blue filled diamonds), *yabG *(red filled triangles), and *spsF *(black filled triangles). **(e) ***spoIIA *operon: *spoIIAA *(black filled diamonds), *spoIIAB *(red filled triangles), and *sigF *(open blue circles). **(f) ***spoIIG *operon and *sigG*: *spoIIGA *(green filled diamonds), *sigE *(black filled triangles), and *sigG *(open red squares). The gray bar indicates the onset of transitional phase. **(g) **Ranked expression intensities. White denotes a rank of 1, while dark blue denotes a rank of 100 (see scale). Gray squares indicate timepoints at which the intensity did not exceed the threshold value. Bracketed genes are predicted to be coexpressed as an operon.

**Figure 4:**
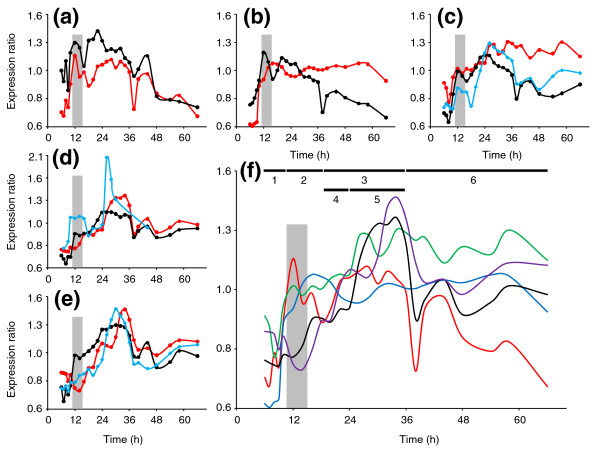
Transcriptional and putative activity profiles for the major sporulation factors. The standardized expression ratios compared to the RNA reference pool of **(a) ***sigH*, **(b) ***spo0A*, **(c) ***sigF*, **(d) ***sigE*, and **(e) ***sigG* are shown in black, while the activity profiles based on the averaged standardized profiles of canonical genes under their control are shown in red. Putative genes (based on the *B. subtilis *model) responsible for activating σ^F ^(*spoIIE*), σ^E ^(*spoIIR*), and σ^G ^(*spoIIIA *operon) are shown as light blue diamonds. For the *spoIIIA *operon, the individual standardized ratios (Figure S13g in Additional data file 4) were averaged together. The gray bar indicates the onset of the transitional phase. **(f) **Compilation of the activity profiles for *sigH *(red), *spo0A *(blue), *sigF *(green), *sigE *(black), and *sigG *(purple). The numbers along the top correspond to the clusters in Figure 1c,d and the bars indicate the timing of each cluster.

Sporulation in bacilli is initiated by a multi-component phosphorelay [27], which is absent in clostridia, but the master regulator of sporulation, Spo0A, is conserved [1,13]. Briefly, in *B. subtilis*, phosphorylated Spo0A promotes the expression of prespore-specific sigma factor σ^F ^and mother cell-specific sigma factor σ^E ^[28]. σ^F ^is followed by σ^G^, which is controlled by both σ^F ^and σ^E^, and σ^E ^is followed by σ^K^, which is controlled by σ^E ^and SpoIIID [28]. *sigH *expression, in bacilli, is induced before the onset of sporulation and aids *spo0A *transcription [28]. Here, *sigH *expression underwent a modest two-fold induction, relative to the first timepoint, during the onset of sporulation but never increased beyond three-fold, in contrast to all other sporulation factors (Figure 3a). *spo0A *expression also peaked during the onset of sporulation at over 12-fold and maintained a minimum of 3-fold induction until hour 36 (Figure 3a,b). Once phosphorylated, in bacilli and likely in *C. acetobutylicum *[29], Spo0A regulates the expression of the operons encoding *sigF*, *sigE*, and *spoIIE *[30], the latter of which acts as an activator of σ^F^. *sigF *and *sigE *exhibited an initial 16- and 8-fold induction, respectively, at hour 12, the timing of peak *spo0A *expression, but a second higher level of induction, 46- and 66-fold, respectively, was reached later at hour 24 (Figure 3c) and confirmed with Q-RT-PCR (Figure 2). The plateau or decrease in expression of *spo0A*, *sigF*, and *sigE *coincided with the peak expression of two known repressors, *abrB *and *sinR*, of sporulation genes in *B. subtilis *(Figure 3b), the former repressing the expression of *spo0A *promoters and the latter directly binding to the promoter sequences of the *spo0A*, *sigF*, and *sigE *operons [31,32]. *C. acetobutylicum *contains three paralogs of *abrB*, among which CAC0310 exhibited the highest promoter activity and, when downregulated, causes delayed sporulation and decreased solvent formation [33]. *sinR *(CAC0549) expression in *C. acetobutylicum *was previously reported [33] to be weak, but our data show a significant amount of expression and suggest a similar role as that in *B. subtilis*. In *B. subtilis*, Spo0A either indirectly (*sinR*) or directly (*abrB*) represses the genes of these two repressors [32,34]. The expression patterns of both genes did decrease after peak Spo0A~P deduced activity (Figure 4b; see below), indicating a similar regulatory network may be involved in *C. acetobutylicum*. *sigF*, *sigE *and *sigG *have very similar expression patterns (Figure 3c). Both *sigF *and *sigE *are activated by Spo0A~P, so similar expression profiles were expected. In *B. subtilis*, a *sigG *transcript is also detected early, but this transcript is read-through from *sigE*, located immediately upstream of *sigG*, and is not translated [35,36]. Translation of *sigG *occurs when the gene is expressed as a single cistron from a σ^F^-dependent promoter located between *sigE *and *sigG *[35,36]. In *C. acetobutylicum*, *sigE *and *sigG *are also located adjacent to each other, but a σ^F ^promoter was not predicted between the two genes [37]. Thus, it was predicted that *sigG *is only expressed as part of the *sigE *operon (consisting of *spoIIGA*, the processing enzyme for σ^E^, and *sigE*). Our transcriptional data seem to support this prediction because all three genes, *spoIIGA*, *sigE*, and *sigG*, have very similar transcriptional patterns (Figure 3f), suggesting they are expressed as a single transcript, like the *spoIIAA*-*spoIIAB*-*sigF *operon (Figure 3e). However, from Northern blots probing against *sigE*-*sigG*, three separate transcripts were seen: one for *spoIIGA*-*sigE*-*sigG*, one for *spoIIGA*-*sigE*, and one for *sigG *[29]. Unfortunately, the current data cannot resolve this issue definitively, since the microarrays only detect if a transcript is present or not.

#### Deduced activity profiles of sporulation factors

We also desired to estimate the activity profiles for the key sporulation factors (σ^H^, Spo0A, σ^F^, σ^E^, and σ^G^; Figure 4). We did so by averaging the expression profiles of known or robustly identifiable canonical genes of their regulons [1]. To adjust for differences in relative expression levels, expression profiles were standardized before averaging [7]. This is a surrogate reporter assay, which we believe is as accurate as most reporter assays. For a detailed discussion of the genes used to construct the plots, see Additional data file 4. For all of the plots (Figure 4), peak activity took place after peak expression, as expected. Of all the factors, σ^H ^activity peaked first, during early transitional phase, and this was followed by a decrease in activity until stationary phase, when activity increased again (Figure 4a,f). Spo0A~P activity was the next to peak, during late transitional phase, and stayed fairly constant throughout the rest of the timecourse (Figure 4b,f). σ^F ^activity had an initial induction during transitional phase, but then stayed constant until 24 hours (Figure 4c,f). After 24 hours, the activity increased again and stayed fairly constant at this higher activity level for the rest of the culture. σ^E ^activity increased slightly during late transitional phase, but its major increase occurred after 24 hours during mid-stationary phase (Figure 4d,f). Like the previous sigma factors, σ^G ^activity increased throughout early stationary phase and early mid-stationary phase, but the major increase occurred after hour 30 (Figure 4e,f). The activity of all of the factors, except for Spo0A and σ^F^, decreased during late stationary phase at hour 38. σ^G ^activity began to increase slightly again at hour 48 but did not peak again. Considering only major peaks in activity, the *Bacillus *model of sporulation is generally true with the peaks progressing from σ^H ^to Spo0A~P to σ^F ^to σ^E ^and finally to σ^G ^(Figure 4f).

#### Can we deduce the activation and processing of σ^F^, σ^E^, and σ^G ^from transcriptional data?

In *B. subtilis*, the sigma factors downstream of Spo0A (σ^F^, σ^E^, and σ^G^) are all regulated by a complex network of interactions [1]. We desired to examine if our transcriptional data could be used to do a first test to determine whether the mechanisms employed in the *B. subtilis *model are valid for *C. acetobutylicum*. In *B. subtilis*, σ^F ^is held inactive in the pre-divisional cell by the anti-σ^F ^factor SpoIIAB. σ^F ^is released when the anti-anti-σ^F ^factor SpoIIAA is dephosphorylated by SpoIIE, resulting in SpoIIAA binding to SpoIIAB, which then releases σ^F^. In *C. acetobutylicum*, *spoIIAB *(CAC2307) and *spoIIAA *(CAC2308) are transcribed on the same operon as *sigF *(Figure 3e), but *spoIIE *(CAC3205) is transcribed separately. The initial increase in σ^F ^activity during the transitional phase was not accompanied by an increase in *spoIIE *expression, but the peak in σ^F ^activity did occur after *spoIIE *upregulation (Figure 4c). Despite the sustained level of σ^F ^activity, *sigF *and *spoIIE *decreased in expression, though *spoIIE *expression did increase slightly again after 48 hours (Figure 4c). In *B. subtilis*, the pro-σ^E ^translated from the *sigE *gene undergoes processing from SpoIIGA, which must interact with SpoIIR in order to accomplish the σ^E ^activation. In *C. acetobutylicum*, SpoIIGA (CAC1694) is transcribed on the same operon as *sigE *(Figure 3f), and SpoIIR is coded by CAC2898. σ^E ^activity increased with the induction of *spoIIR *(Figure 4d), suggesting a similar mechanism as in *B. subtilis*. Finally, σ^G ^activation in *B. subtilis* is dependent upon the eight genes within the *spoIIIA *operon. Here, the second and larger increase in σ^G ^activity followed peak expression of the *spoIIIA *operon, but the early increase in σ^G ^activity was not characterized by a large induction of *spoIIIA *expression (Figure 4e). We tentatively conclude that the *B. subtilis *processing and activation model does generally hold true in *C. acetobutylicum*, but further investigation is needed to determine the exact timing and interaction of the various factors and their activators.

#### Is there a functional *sigK*?

In *B. subtilis*, σ^K ^is formed by splicing together two genes (*spoIVCB *and *spoIIIC*), both under the control of σ^E ^and SpoIIID [38], separated by a *skin *element [39]. In contrast, a single gene encoding σ^K ^has been annotated in *C. acetobutylicum *[13]. The gene was initially identified using a PCR-approach [40] and was later detected by primer extension in a phosphate-limited, continuous culture of *C. acetobutylicum *DSM 1731 [41]. *spoIIID*, which controls *sigK *expression with σ^E ^in *B. subtilis*, reached peak expression at hour 30, which is consistent with it being under σ^E ^control (Figure 3d) [42]. However, at no timepoint in this study did *sigK *exceed the cutoff expression criterion. Q-RT-PCR also showed a significantly lower *sigK *induction compared to the other sigma factors and suggests the transcript, if expressed, is at much lower levels than any other gene analyzed (Figure 2). The putative main σ^K ^processing enzyme, SpoIVFB (CAC1253), also did not exceed the cutoff criterion. To help determine if there is an active σ^K^, we investigated two genes controlled by σ^K ^in *B. subtilis*. *yabG *(CAC2905), which encodes a protein involved in spore coat assembly, was upregulated mid-stationary phase and peaked at hour 30 (Figure 3d), and *spsF *(CAC2190), involved in spore coat synthesis, was not upregulated until late stationary phase, at hour 38 (Figure 3d). From these two genes, it is difficult to determine whether a functional *sigK *gene exists or not. Clearly they are both transcribed, but based on its expression pattern, *yabG *could fall under the control of σ^E ^instead of σ^K^. *spsF *upregulation is late enough to possibly indicate σ^K ^regulation though. Ideally, more genes need to be investigated to draw firmer conclusions, but because few σ^K ^regulon homologs exist in *C. acetobutylicum*, we cannot currently determine if there is σ^K ^activity or not.

### Distinct profiles of sensory histidine kinases: which for Spo0A?

#### Revisiting the orphan kinases

As discussed, phosphorylated Spo0A is responsible for initiating sporulation in both bacilli and clostridia along with solvent formation in *C. acetobutylicum*. In bacilli, Spo0A is phosphorylated via a multi-component phosphorelay [43], initiated by five orphan histidine kinases, KinA-E (kinases that lack an adjacent response regulator); this phosphorelay system is absent in all sequenced clostridia [1]. Alternatively, Spo0A in clostridia may be directly phosphorylated by a histidine kinase, orphan or not, as was hypothesized in [1,7]. This alternative was demonstrated in *C. botulinum*, where the orphan kinase CBO1120 was able to phosphorylate Spo0A [44]. In *C. acetobutylicum*, five true orphan kinases have been identified with a sixth orphan, CAC2220, identified as CheA, which has a known response regulator [1].

**Figure 5:**
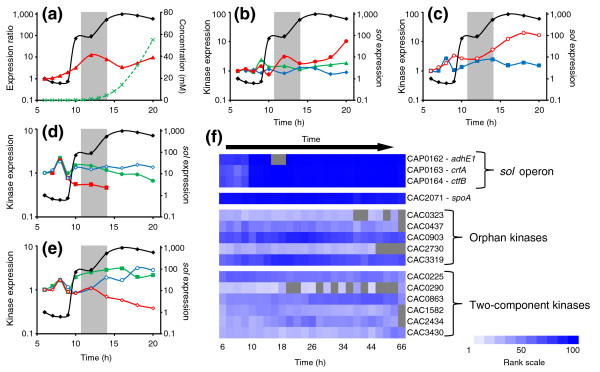
Expression profiles of uncharacterized sensory histidine kinases that could phosphorylate Spo0A. Gene and operon profiles are ratios compared against the first expressed timepoint. Gray bar indicates the onset of the transitional phase. **(a) **Activation of Spo0A as represented through the upregulation of the *sol *operon (black filled diamonds; CAP0162-164) and the production of butanol (green crosses). Activation occurs before *spo0A *(red filled triangles) reaches peak expression. **(b) **Expression of the orphan kinases CAC0323 (blue filled diamonds), CAC0437 (green filled triangles), and CAC0903 (red filled circles) relative to the *sol *operon (black filled diamonds) (right-hand side vertical axis). **(c) **Expression of the orphan kinases CAC2730 (blue filled squares) and CAC3319 (open red circles) relative to the *sol *operon (black filled diamonds) (right-hand side vertical axis). **(d) **Expression of the two-component kinases CAC0225 (green filled circles), CAC0290 (red filled squares), and CAC0863 (open blue diamonds) relative to the *sol *operon (black filled diamonds) (right-hand side vertical axis). **(e) **Expression of the two-component kinases CAC1582 (green filled squares), CAC2434 (open blue circles), and CAC3430 (open red diamonds) relative to the *sol *operon (black filled diamonds) (right-hand side vertical axis). **(f) **Ranked expression intensities. White denotes a rank of 1, while dark blue denotes a rank of 100 (see scale). Plot covers the entire timecourse, whereas the previous figures only covered the first 14 hours. Gray squares indicate timepoints at which the intensity did not exceed the threshold value.

A kinase that could directly phosphorylate Spo0A is expected to have a peak in expression before or during the activation of Spo0A, as the orphan kinases in *B. subtilis *do [45-47]. As a measure of Spo0A activity, the expression of the *sol *operon (CAP0162-64) was used, as before [7], because it is induced by Spo0A~P. The initial induction of the *sol *operon, almost 100-fold, occured at hour 10 (before *spo0A *reached it maximum expression), with detectable levels of butanol appearing before the second induction of the *sol *operon. This second induction, of another 10-fold, followed the peak in *spo0A *expression (Figure 5a). It is clear that some level of phosphorylated Spo0A exists at 10 hours; therefore, kinase candidates must display an increase in expression before 10 hours. Of the five orphan kinases (Figure 5b,c), CAC2730 displayed the earliest peak followed by CAC0437, CAC0903, and CAC3319. CAC0323 never displayed a prominent peak in expression either before or after *sol *operon induction (Figure 5b) and likely does not play a role in phosphorylating Spo0A. Of the remaining four, CAC0437 and CAC2730 peaked only once before the initial *sol *operon induction, while CAC0903 peaked before each induction of the *sol *operon (Figure 5b,c). CAC3319 expression slightly mirrored that of the *sol *operon, with an increase before initial induction followed by a plateau, and an increase in expression again until it peaked just after the *sol *operon peaked (Figure 5c). The proteins encoded by CAC0437 and CA0903 displayed the most similarity to the protein encoded by CBO1120, the orphan kinase in *C. botulinum *shown to phosphorylate Spo0A [44].

#### Non-orphan kinase expression

Though primarily interested in orphan kinases because of the similarity to the *B. subtilis *model, a two-component response system could also be responsible for the phosphorylation of Spo0A. The remaining 30 annotated histidine kinases were also investigated to determine if any displayed a peak in expression before the initial induction of the *sol *operon (Additional data file 5). Six kinases (Figure 5d,e) were found to have a peak in expression at 8 hours. CAC0290 and CAC3430 subsequently decreased in expression while CAC0225 and CAC0863 maintained expression at initial levels. Despite a dip in expression at hour 9, CAC1582 maintained an increased expression level from 8 hours on. CAC2434 peaked at hour 8, dropped back to initial levels, but then steadily increased with the second induction of the *sol *operon.

### Sigma factors of unknown function: a first assessment of their functional roles

**Figure 6:**
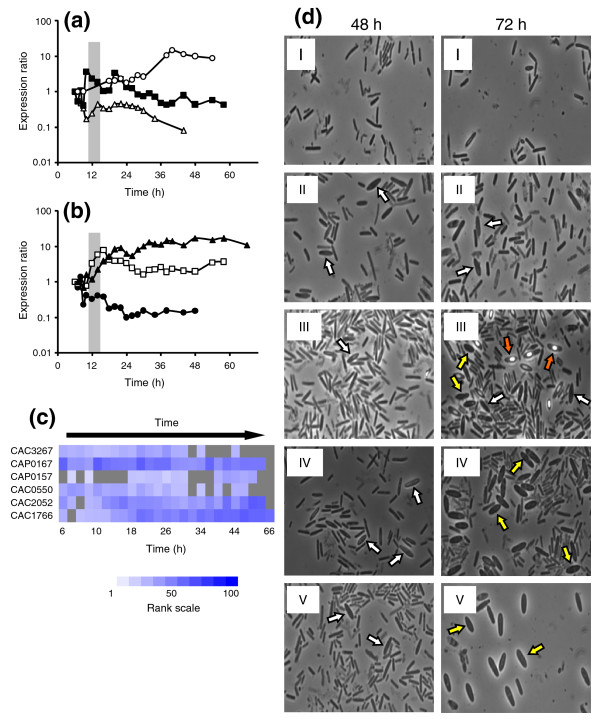
Expression profiles of sigma factors with unknown function and the effects of down-regulation. **(a) **Expression profiles of CAC3267 (open triangles), CAP0167 (filled squares), and CAP0157 (open circles) as ratios compared to the first expressed timepoint. Gray bar indicates the onset of transitional phase. **(b) **Expression profiles of CAC0550 (filled circles), CAC2052 (open squares), and CAC1766 (filled triangles) as ratios compared to the first expressed timepoint. Gray bar indicates the onset of transitional phase. **(c) **Ranked expression intensities of the sigma factors. White denotes a rank of 1, while dark blue denotes a rank of 100 (see scale). Gray squares indicate timepoints at which the intensity did not exceed the threshold value. **(d) **Microscopy time-course of asRNA strains compared to WT and plasmid control strains. Microscopy samples from WT (I) and pSOS95del (II) cultures (as controls) and three asRNA strains taken for two timepoints over a course of 72 hours. At 72 hours, WT (I) and pSOS95del (II) exhibit the typical clostridial forms (white arrows), while asCAP0166 (III) shows advanced differentiation with forespores and endospores (orange arrows) already visible. Strains asCAP0166 (III), asCAP0167 (IV), and asCAC1766 (V) show a novel, extra-swollen clostridial form (yellow arrows).

Seventeen sigma factors are annotated on the *C. acetobutylicum *genome, including two on pSOL1. Two, *sigK *(CAC1689) and CAC1770 (a *sigK*-like sigma factor), are expressed at very low levels and two others, CAC1509 (annotated 'specialized sigma subunit of RNA polymerase') and CAC1226 (one of two annotated *sigA*s), are only above the expression cutoff in 8 out of 25 timepoints, and these timepoints are not consecutively expressed. Among the expressed sigma factors, six, CAP0157, CAP0167, CAC3267, CAC1766, CAC2052, and CAC0550, are of unknown function, while the remaining seven expressed sigma factors (σ^H^, σ^F^, σ^E^, σ^G^, σ^A^, σ^D^, and σ^54^/rpoN) are of predicted known function. To assess the potential role of the remaining six sigma factors of unknown function, we examined the transcriptional profiles (Figure 6a,b) and probed the binding motifs in their promoter regions for predicted Spo0A, σ^A^, σ^E^, and σ^F^/σ^G ^binding motifs [37].

#### Transcriptional analysis of the sigma factors of unknown function

Loss of pSOL1 impairs sporulation at the level of *spo0A *expression [7,48], thus generating increased interest for sigma factors located on the pSOL1 plasmid as these may play a role in the regulation of sporulation. Two sigma factors, CAP0157 and CAP0167, are located on pSOL1 and are annotated as 'special sigma factor (σ^F^/σ^E^/σ^G ^family)' and 'specialized sigma factor (σ^F^/σ^E ^family)', respectively. It was predicted that CAP0167 is putatively co-transcribed with CAP0166 from a promoter of the σ^F^/σ^G ^family [37] and it displayed an expression pattern similar to that of *spo0A*, consistent with the computational prediction of an 0A box [29] and two reverse 0A boxes in its promoter region (Figure 6a). CAP0157 was expressed from an unidentified promoter late in the timecourse (40+ hours) and thus may be involved in late-stage sporulation, despite its low level of expression at hour 20 (Figure 6a). CAC3267, putatively the fourth gene in an operon starting with CAC3270 and ending with CAC3264 [37], was mainly expressed during early exponential growth (Figure 6a), then decreased, and peaked again around 14 hours, after which expression decreased again. This pattern of expression suggests that it plays a role in vegetative growth and possibly early sporulation. CAC0550, putatively transcribed from a σ^A ^promoter as a single cistron [37], was mainly transcribed early with its expression ending after 20-24 hours (Figure 6b), suggesting that it is not involved in sporulation. CAC1766, expressed from an unknown promoter, displayed a unique pattern with a progressive buildup starting around hours 8-12 and a distinct peak around hour 22 (Figure 6b). CAC2052 is annotated as 'DNA-dependent RNA polymerase σ-subunit' and was putatively expressed together with CAC2053, a hypothetical protein, from a σ^A ^and/or a σ^F^/σ^G ^promoter [37]. Our data suggest that it is unlikely to be transcribed from a σ^F^/σ^G ^promoter without any other effectors, as their transcription peaked at hour 16, when there was very little (if any) σ^F ^or σ^G ^activity (Figure 6b).

#### Phylogenetic tree comparison

To help determine a possible function for these sigma factors, a phylogenetic tree was constructed of σ^70 ^sigma factors from ten species, including *B. subtilis *and all sequenced clostridial species. The resulting tree (Additional data file 6) contains eleven major branches, and of these, seven can be definitively classified based on known sigma factors within the branch. These categories are extracytoplasmic function (ECF), sporulation factors (*sigF*, *sigE*, and *sigG*), *sigH*, *sigA *(a basal sigma factor), *sigD *(regulates chemotaxis and motility), and *sigB *(a general response sigma factor). Two factors, CAC3267 and CAC1766, fell within ECF branches. CAC3267 fell within an ECF branch close to the *B. subtilis* σ^V^, a sigma factor of unknown function, and σ^M^, a sigma factor essential for growth and survival in high salt concentrations. CAC1766 fell within a different ECF branch close to *B. subtilis* σ^Z^, a sigma factor of unknown function, and CAC1509, a sigma factor expressed for less than eight consecutive timepoints. The remaining four factors fell within clusters with other clostridial sigma factors of unknown function, though several could have possible ECF function.

#### Antisense RNA knock-down of four sigma factors: 'fat' clostridial forms and enhanced glucose metabolism

Of the six expressed sigma factors of unknown function, CAP0157, CAP0167, CAC2052, and CAC1766 were chosen for further study because the timing and shape of their expression patterns suggested potential involvement in sporulation and/or solventogenesis. Since the two processes are coupled, phenotypic changes in differentiation may affect solvent production, as has been previously observed [4,6,29,33,49]. Antisense RNA (asRNA) knock-down was chosen over knocking out the genes, because knockouts are still extremely difficult to produce in this and all other clostridia. Indeed, to date, only a handful of knockouts have been created [29,50-53], and these have only been achieved after screening thousands of transformants [51-53]. Recently, a group II intron system has been developed for clostridia [54], but this system was not yet available when these experiments were carried out. In contrast, asRNA is relatively quick, has been shown to reduce gene expression by up to 90% [33,55,56] and has been used to knock-down a large number of genes with a high level of specificity [33,49,55-59]. asRNA constructs (see Additional data file 7 for specific sequences used) were designed against CAP0157, CAP0167, CAC2052, and CAC1766 along with CAC2053 and CAP0166, the first genes in the operons predicted to contain CAC2052 and CAP0167, respectively [37]. Cultures of these strains were examined and compared against the wild type (WT) and plasmid control strain 824(pSOS95del) for cell morphology differences and metabolic changes.

Microscopy results from the asRNA-strain cultures revealed both novel morphologies and apparently altered differentiation (Figure 6d). Most notable were changes in strains asCAP0166, asCAP0167 and asCAC1766. Typical WT cultures display a predominately vegetative, symmetrically dividing population through 72 hours as evidenced by the thin, rod-shaped, phase dark cells (Figure 6d, I). By 72 hours, WT cultures exhibited only a small percentage of swollen, cigar-shaped clostridial forms and then a proportional population of free spores by 96 hours.

pSOS95del cultures exhibited clostridial forms by 48 hours, suggesting an accelerated differentiation compared to WT, as has been seen before in our laboratory (Figure 6d, II). Moreover, a greater percentage of clostridial forms and free spores compared to WT were observed at 72 and 96 hours, respectively. asCAP0166 cultures generated a large percentage of clostridial forms and endospores/free spores by hours 48 and 72, respectively (Figure 6d, III). This differentiation is accelerated in comparison to pSOS95del. By hour 96, asCAP0166 cultures exhibited predominately vegetative cells apparently derived from germinated spores (data not shown). asCAP0167 cultures also exhibited accelerated differentiation and displayed a novel (to our knowledge) form of cellular morphology that was most profoundly observable at 72 hours (Figure 6d, IV). This novel morphology has qualities of an excessively swollen clostridial cigar-form (which makes them look much shorter than normal clostridial forms), with what appears to be endospore formation occurring, but without the associated phase bright characteristics seen in the 72 hour asCAP0166 cultures. The asCAP0166 culture displayed cells in this novel morphological state as well, but to a lesser extent, although it is possible that because of its faster sporulation, such cell forms appeared prior to 72 hours. The asCAC1766 cultures also exhibited altered differentiation; most importantly, at 72 hours the majority of the cells exhibited a very swollen clostridial-form morphology similar to that in the asCAP0167 cultures at 72 hours, but slightly more elongated (Figure 6d, V).

**Figure 7:**
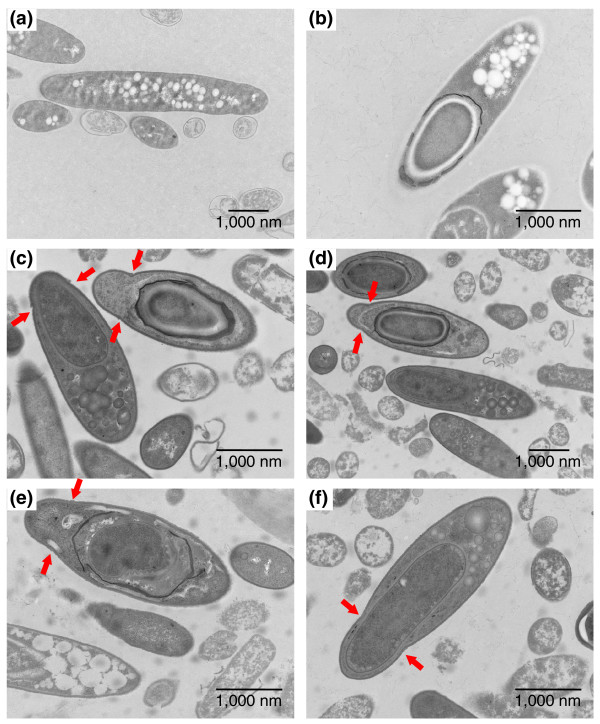
TEM images of the novel cell forms. **(a-b) **TEM images of the plasmid control strain pSOS95del: typical elongated clostridial form with electron translucent granulose (a); typical endospore form with a developing endospore at one end of the cell and electron translucent granulose still visible at the other end of the cell (b). **(c-d) **TEM images of the antisense strain asCAP0167. **(e-f) **TEM images of the antisense strain asCAC1766. Red arrows in (c-f) indicate pinched portions of the cell membrane not seen in the control strain and are characteristic of this novel cell type. Also noticeable is the electron dense granulose in the antisense strains, in contrast to the electron translucent granulose in the control samples.

**Figure 8:**
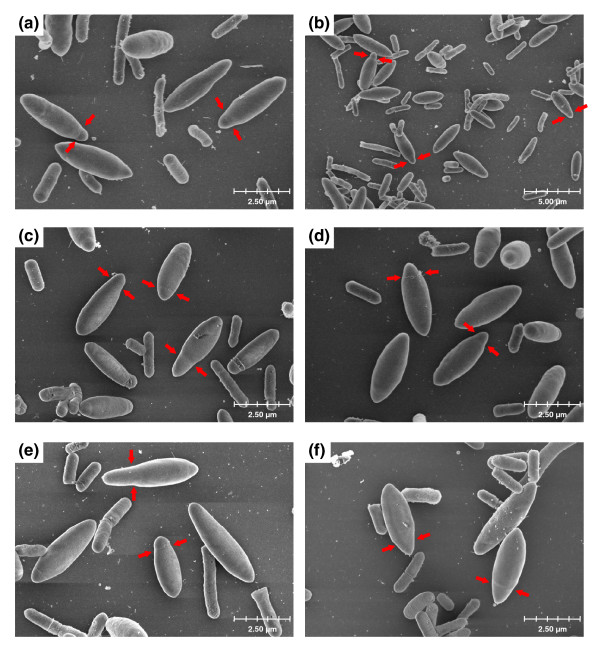
Scanning electron microscopy (SEM) images of the novel cell forms. SEM images of the antisense strains **(a-c) **asCAP0167 and **(d-f) **asCAC1766. Red arrows in indicate pinched portions of the cell membrane not seen in the control strain and are characteristic of this novel cell type.

To further characterize this novel cell form, transmission electron microscopy (TEM) and scanning electron microscopy images of cells were taken for strains asCAP0167 and asCAC1766. To determine morphological differences involved in differentiation, the TEM images were compared against cell images taken from the plasmid control strain (Figure 7). For both asRNA strains, the very swollen cell forms observed can be documented as approximately 2.5-4 μm long, and 1.1-1.3 μm in diameter, and should be compared to control or WT swollen clostridial forms, which are 3.5-6 μm long and 0.8-1 μm in diameter. Forespore and endospore forms of both asCAP0167 (Figure 7c,d) and asCAC1766 (Figure 7e,f) displayed a pinched end not seen in the plasmid control (Figure 7b). A slight pinching is seen in the clostridial forms of the plasmid control strain (Figure 7a), but this is probably indicative that an asymmetric division is about to occur. Rather, the pinched ends seen in the antisense strains occur after asymmetric division and while the spore is developing within the mother cell. These pinched ends are also noticeable in the scanning electron microscopy images (Figure 8). Though granulose is distinguishable in most of the TEM images (Figure 7c,d,f), it is not the characteristic electron translucent seen in typical clostridial, forespore, and endospore forms (Figure 7a,b). These differences were seen throughout the culture and additional TEM images of both the plasmid control and the antisense strains are included in Additional data file 8.

Glucose, acetone, and butanol concentrations from two to four biological replicates for each strain were averaged together, and the results are shown in Table 1. We averaged data from cultures that displayed similar characteristics; most cultures did so despite the fact that each culture was inoculated from a different colony for each strain. Acetone and butanol levels were typical for WT and control cultures, with the WT producing 90 mM of acetone and 150 mM of butanol and the plasmid-control strain producing 80 mM of acetone and 160 mM of butanol [60]. By 192 hours, all strains had either produced comparable amounts of butanol to the WT and the plasmid control strain or had somewhat outperformed these two strains. The most significant differences were that all asRNA strains consumed higher levels of glucose and also had a delayed metabolism in terms of product formation. These metabolic changes, although preliminary, are consistent with and support the large changes in the kinetics of sporulation observed by microscopy.

**Table 1:** Concentrations of glucose, acetone, and butanol for asRNA strains

	96 hours			144 hours			192 hours*		
			
Sample	Glucose^†^	Acetone^†^	Butanol^†^	Glucose^†^	Acetone^†^	Butanol^†^	Glucose^†^	Acetone^†^	Butanol^†^
Wild type	165	91	157	143	74	157	120	61	162
pSOS95del^‡^	264	57	97	136	83	169	125	57	158
asCAC1766	274	67	84	118	123	169	114	97	163
asCAC2052	294	49	69	191	84	122	116	92	154
asCAC2053	285	54	77	158	94	142	94	88	161
asCAP0157	314	49	63	198	91	122	96	111	174
asCAP0166	290	55	77	118	125	167	77	91	176
asCAP0167	294	54	73	78	125	180	56	98	185

*At 192 hours, significant amounts of acetone had evaporated along with small amounts of butanol. However, the cultures were still metabolically active, as indicated by the decreased amounts of glucose and increased amounts of butanol. ^†^Concentrations are mM. ^‡^pSOS95del was used as a plasmid control strain.

## Conclusion

This detailed and previously unrevealed transcriptional roadmap has allowed for the first time a complete investigation of the genetic events associated with clostridial differentiation. We were able to link distinct and striking global transcriptional changes to previously known important morphological and physiological changes. To date, this is the most complete genetic analysis of the different morphological forms: vegetative, clostridial, and forespore/endospore. Importantly, this analysis was performed on a mixed culture, which may either dilute or produce noise in the data, but investigation of the clusters identified revealed that these clusters do capture important known processes. We were also able to identify a cell population late in the timecourse similar to vegetative cells. Visually, these late cells looked and acted like vegetative cells, and transcriptionally, they were also fairly similar. The major cell motility and chemotaxis genes were upregulated both early and late in the timecourse (Figure S2 in Additional data file 3), as were the ribosomal proteins (Figure S12 in Additional data file 3). Also, the cell division associated genes *rodA*, *ftsE*, and *ftsX *follow the same transcriptional pattern of both early and late expression (Figure S11 in Additional data file 3). Although, these cells stain differently from the early vegetative cells, probably due to changes in membrane structure in response to the presence of solvents and do not produce detectable levels of acids or solvents, we believe these cells are germinated cells from spores produced early in the timecourse. While the triggers for both sporulation and germination are not known [1], the culture late in the timecourse is less acidic because of the acid reassimilation, and pH has been shown to be a trigger for sporulation [21].

This study has also allowed the first full comparison to the widely studied *B. subtilis *sporulation program. We have confidently identified the temporal orchestration of all known sporulation-related transcription factors and conclude the *Bacillus *model generally holds true with the cascade progressing in the following manner: σ^H^, Spo0A, σ^F^, σ^E^, and σ^G ^(Figure 4f). In addition, we can conclude that the major activating/processing proteins involved in sigma factor activation in *B. subtilis *play a similar role in *C. acetobutylicum*, though additional investigation is needed to clarify their role. Of significance is the lack of *sigK *signal. The genes responsible for transcribing *sigK *in *B. subtilis*, *sigE *and *spoIIID*, were expressed, but the putative processing enzyme *spoIVFB *was not. Two genes under the control of σ^K ^in *B. subtilis *were expressed, but their expression patterns are not consistent with each other. Based on the expression pattern of *yabG*, it could be controlled by σ^E^, while the late expression of *spsF *could be an indication of σ^K ^activity.

Finally, in order to determine if one of the annotated sigma factors of unknown function could be a *sigK*-like gene, we first investigated their transcriptional profiles. CAP0157 was a possible candidate with its upregulation late in the timecourse, as was CAC1766 since its expression was sustained throughout the stationary phase (Figure 6a,b). Neither of these genes, nor any of the other sigma factors of unknown function, clustered close to the known sporulation-related sigma factors on the phylogenetic tree (Additional data file 6), but when downregulated using asRNA, both CAC1766 and the CAP0167 operon (CAP0166 and CAP0167) displayed altered differentiation (Figures 6d, 7 and 8). Though involved in differentiation, the exact role of these two sigma factors is difficult to assess because of the incomplete silencing of the genes through asRNA downregulation. Mature free spores and typical endospore forms without a pinched end are still seen (data not shown), but whether these develop from the novel cell types or from cells not affected by the antisense cannot be determined. Interestingly, both CAP0167 and CAC1766 clustered together with other clostridial sigma factors and closer to ECF sigma factors than to the major sporulation sigma factors *sigF*, *sigE*, and *sigG *(Additional data file 6). In *B. subtilis*, ECF sigma factors do not play a role in differentiation [61,62], though a triple mutant in *sigM*, *sigW*, and *sigX *did display altered phenotypes [62]. The fact that CAC1766 and CAP0167 appear to affect the developmental process of sporulation (Figures 7 and 8; Additional data file 8) suggests either that ECF factors may play a role in sporulation in clostridia or that a novel category of sigma factors exist in clostridia that play a role in sporulation.

## Materials and methods

### Fermentation analysis

Two cultures of *C. acetobutylicum *ATCC 824 were grown in pH controlled (pH >5) bioreactors (Bioflow II and 110, New Brunswick Scientific, Edison, NJ, USA) [7]. Cell density, substrate and product concentrations were analyzed as described [56].

### RNA isolation and cDNA labeling

Samples were collected by centrifuging 3-10 ml of culture at 5,000×g for 10 minutes, 4°C and storing the cell pellets at -85°C. Prior to RNA isolation, cells were washed in 1 ml SET buffer (25% sucrose, 50 mM EDTA [pH 8.0], and 50 mM Tris-HCl [pH 8.0]) and centrifuged at 5,000×g for 10 minutes, 4°C. Pellets were processed similarly to [7] but with the noted modifications. Cells were lysed by resuspending in 220 μl SET buffer with 20 mg/ml lysozyme (Sigma, St. Louis, MO, USA) and 4.55 U/ml proteinase K (Roche, Indianapolis, IN, USA) and incubated at room temperature for 6 minutes. Following incubation, 40 mg of acid-washed glass beads (≤106 μm; Sigma) were added to the solution, and the mixture was continuously vortexed for 4 minutes at room temperature. Immediately afterwards, 1 ml of ice cold TRIzol (Invitrogen, Carlsbad, CA, USA) was added; 500 μl of sample was diluted with an equal volume of ice cold TRIzol and purified. Following dilution, 200 μl of ice cold chloroform was added to each sample, mixed vigorously for 15 s, and incubated at room temperature for 3 minutes. Samples were then centrifuged at 12,000 rpm in a tabletop microcentrifuge for 15 minutes at 4°C. The upper phase was saved and diluted by adding 500 μl of 70% ethanol. Samples were then applied to the RNeasy Mini Kit (Qiagen, Valencia, CA, USA), following the manufacturer's instructions. To minimize genomic DNA contamination, samples were incubated with the RW1 buffer at room temperature for 4 minutes. The method disrupted all cell types equally, as evidenced by microscopy (data not shown). cDNA was generated and labeled as described [7]. The reference RNA pool contained 25 μg of RNA from samples taken from the same culture at 8, 10, 12, 14, 16, 18, 20, 22, 24, 26, 28, 30, 32, 34, 36, 38, 40, 44, 48, 54, 58, and 66 h.

### Microarray analysis

Agilent technology 22k arrays, (GEO accession number GPL4412) as described in [63], were hybridized, washed, and scanned per Agilent's recommendations. Spot quantification employed Agilent's eXtended Dynamic Range technique with gains of 100% and 10% (Agilent's Feature Extraction software (v. 9.1)). Normalization and slide averaging was carried out as described [7,63]. A minimum intensity of 50 intensity units was used as described [63]. Microarray data have been deposited in the Gene Expression Omnibus database under accession number GSE6094. To gain a qualitative measure of the abundance of an mRNA transcript, the averaged normalized log mean intensity values were ranked on a scale of 1 (lowest intensity value) to 100 (highest intensity value). Genes were clustered using TIGR's MEV program [64].

### Quantitative RT-PCR

Q-RT-PCR was performed as described [48]. Specific primer sequences are included in Additional data file 9; CAC3571 was used as the housekeeping gene.

### Microscopy

For light microscopy, samples were stored at -85°C after 15% glycerol was added to the sampled culture. Samples were then pelleted, washed twice with 1% w/v NaCl and fixed using 50 μl of 0.05% HCl/0.5% NaCl solution to a final count of 10^6 ^cells/μl. Slides were imaged using a Leica widefield microscope with either phase contrast or Syto-9 and PI dyes (Invitrogen LIVE/DEAD *Bac*Light Kit) to distinguish cell morphology.

For electron microscopy, samples were fixed by addition of 16% paraformaldehyde and 8% glutaraldehyde to the culture medium for a final concentration of 2% paraformaldehyde and 2% glutaraldehyde. For cultures grown on plates, colonies were scraped from the agar and suspended in 2% paraformaldehyde and 2% glutaraldehyde in 0.1 M sodium cacodylate buffer (pH 7.4). Cultures were fixed for 1 h at room temperature, pelleted and resuspended in buffer.

For transmission electron microscopy, bacteria were pelleted, embedded in 4% agar and cut into 1 mm × 1 mm cubes. The samples were washed three times for 15 minutes in 0.1 M sodium cacodylate buffer (pH 7.4), fixed in 1% osmium tetroxide in buffer for 2 h, and then washed extensively with buffer and double de-ionized water. Following dehydration in an ascending series of ethanol (25, 50, 75, 95, 100, 100%; 15 minutes each), the samples were infiltrated with Embed-812 resin in 100% ethanol (1:3, 1:2, 1:1, 2:1, 3:1; 1 h each) and then several changes in 100% resin. After an overnight infiltration in 100% resin, the samples were embedded in BEEM capsules and polymerized at 65°C for 48 h. Blocks were sectioned on a Reichert-Jung UltracutE ultramicrotome and ultrathin sections were collected onto formvar-carbon coated copper grids. Sections were stained with methanolic uranyl acetate and Reynolds' lead citrate [65] and viewed on a Zeiss CEM 902 transmission electron microscope at 80 kV. Images were recorded with an Olympus Soft Imaging System GmbH Megaview II digital camera. Brightness levels were adjusted in the images so that the background between images appeared similar.

For scanning electron microscopy, fixed samples were incubated on poly-L-lysine coated silica wafers for 1 h and then rinsed three times for 15 minutes in 0.1 M sodium cacodylate buffer (pH 7.4). The samples were fixed with 1% osmium tetroxide in buffer for 2 h, washed in buffer and double de-ionized water, and then dehydrated in ethanol (25, 50, 75, 95, 100, 100%; 15 minutes each). The wafers were critical point dried in an Autosamdri 815B critical point drier and mounted onto aluminum stubs with silver paint. The samples were coated with Au/Pd with a Denton Bench Top Turbo III sputter-coater and viewed with a Hitachi 4700 FESEM at 3.0 kV.

### Phylogenetic tree generation

Based on the genome annotations available at NCBI, we considered any sigma factor that was annotated as σ^70 ^or unannotated. A second filter was applied by requiring that all the sequences should contain a Region 2, the most conserved region of the σ^70 ^protein. All members of this class of sigma factor contain Region 2, and it was modeled with the HMM pfam04542. This criterion removed CAC0550, CAC1766 and CAP0157, but they were added to the list again despite their lack of a Region 2. The alignment was made using ClustalW 1.83 using the default settings and visualized as a radial tree as created by Phylodraw v. 0.8 from Pusan National University.

### Generation and characterization of antisense strains

Oligonucleotides were designed to produce asRNA complementary to the upstream 20 bp and first 30-40 bp of the targeted genes' transcripts (Additional data file 7). The constructs were cloned into pSOS95del under the control of a thiolase (*thl*) promoter and confirmed by restriction digest. Plasmids were then methylated and transformed into *C. acetobutylicum *ATCC 824, as previously described [33,55,56]. Strains were grown in 10 ml cultures and characterized using microscopy and HPLC to analyze final product concentrations [56].

## Abbreviations

asRNA, antisense RNA; COG, Cluster of Orthologous Groups; ECF, extracytoplasmic function; PI, propidium iodide; Q-RT-PCR, quantitative reverse transcription PCR; TEM, transmission electron microscopy; WT, wild type.

## Authors' contributions

SWJ carried out the microarray experiments, helped with the electron microscopy, helped analyze the data, and drafted and finalized the manuscript. CJP designed the microarray platform used, helped with the bioinformatic tools used in the analysis, and drafted parts of the manuscript. BT carried out all the microscopy except the electron microscopy and generated the antisense RNA strains. NC carried out the microarray experiments and helped with the generation of the antisense strains. RS helped design the microarray experiments, carried out the Q-RT-PCR experiments, helped analyze the data, and drafted parts of the manuscript. RSS helped with the bioinformatic tools used in the analysis. ETP helped in the design of all the experiments, the analysis and interpretation of the data, and helped in the organization, draft and editing of the manuscript. All authors read and approved the final manuscript.

## Additional data files

The following additional data are available. Additional data file 1 is a figure comparing the present microarray study to an earlier microarray study that examined the early sporulation of *C. acetobutylicum *followed by a brief discussion. Additional data file 2 contains tables detailing the COG analysis for each cluster and all the genes placed in each cluster. Additional data file 3 contains figures of the transcriptional profiles, in terms of both intensity and differential expression, of specific gene clusters with brief discussions following several figures. Additional data file 4 is a composite figure showing the individual expression profiles of the genes that were standardized and averaged and is followed by a brief discussion on how the genes used to construct the deduced activity plots were chosen. Additional data file 5 is a figure showing the differential expression and intensity of all annotated histidine kinases and response regulators. Additional data file 6 is a figure showing the phylogenetic tree resulting from the alignment of the σ^70^-related and unannotated sigma factors from ten bacterial species. Additional data file 7 is a table listing the sequences for each asRNA construct. Additional data file 8 contains figures showing additional TEM images of the plasmid control strain, asCAP0167, and asCAC1766. Additional data file 9 is a table listing the primer sequences used in the Q-RT-PCR experiments.

## Supplementary Material

Additional data file 1 Comparison of the present microarray study to an earlier microarray study that examined the early sporulation of *C. acetobutylicum*. Click here for file

Additional data file 2 COG analysis for each cluster and all the genes placed in each cluster. Click here for file

Additional data file 3 Transcriptional profiles, in terms of both intensity and differential expression, of specific gene clusters. Click here for file

Additional data file 4 Includes a brief discussion on how the genes used to construct the deduced activity plots were chosen. Click here for file

Additional data file 5 Differential expression and intensity of all annotated histidine kinases and response regulators. Click here for file

Additional data file 6 Phylogenetic tree resulting from the alignment of the σ^70^-related and unannotated sigma factors from ten bacterial species. Click here for file

Additional data file 7 Sequences for each asRNA construct. Click here for file

Additional data file 8 TEM images of the plasmid control strain, asCAP0167, and asCAC1766. Click here for file

Additional data file 9 Primer sequences used in the Q-RT-PCR experiments. Click here for file

## References

[B1] Paredes CJ, Alsaker KV, Papoutsakis ET (2005). A comparative genomic view of clostridial sporulation and physiology.. Nat Rev Microbiol.

[B2] Demain AL, Newcomb M, Wu JH (2005). Cellulase, clostridia, and ethanol.. Microbiol Mol Biol Rev.

[B3] Woods DR (1995). The genetic engineering of microbial solvent production.. Trends Biotechnol.

[B4] Alsaker KV, Spitzer TR, Papoutsakis ET (2004). Transcriptional analysis of *spo0A *overexpression in *Clostridium acetobutylicum *and its effect on the cell's response to butanol stress.. J Bacteriol.

[B5] Tomas CA, Beamish J, Papoutsakis ET (2004). Transcriptional analysis of butanol stress and tolerance in *Clostridium acetobutylicum*.. J Bacteriol.

[B6] Zhao Y, Tomas CA, Rudolph FB, Papoutsakis ET, Bennett GN (2005). Intracellular butyryl phosphate and acetyl phosphate concentrations in *Clostridium acetobutylicum *and their implications for solvent formation.. Appl Environ Microbiol.

[B7] Alsaker KV, Papoutsakis ET (2005). Transcriptional program of early sporulation and stationary-phase events in *Clostridium acetobutylicum*.. J Bacteriol.

[B8] Jones DT, Westhuizen A van der, Long S, Allcock ER, Reid SJ, Woods DR (1982). Solvent production and morphological changes in *Clostridium acetobutylicum*.. Appl Environ Microbiol.

[B9] Long S, Jones DT, Woods DR (1983). Sporulation of *Clostridium acetobutylicum *P262 in a defined medium.. Appl Environ Microbiol.

[B10] Comas-Riu J, Vives-Rego J (2002). Cytometric monitoring of growth, sporogenesis and spore cell sorting in *Paenibacillus polymyxa *(formerly *Bacillus polymyxa*).. J Appl Microbiol.

[B11] Yang H, Haddad H, Tomas C, Alsaker K, Papoutsakis ET (2003). A segmental nearest neighbor normalization and gene identification method gives superior results for DNA-array analysis.. Proc Natl Acad Sci USA.

[B12] Tatusov RL, Galperin MY, Natale DA, Koonin EV (2000). The COG database: a tool for genome-scale analysis of protein functions and evolution.. Nucleic Acids Res.

[B13] Nölling J, Breton G, Omelchenko MV, Makarova KS, Zeng Q, Gibson R, Lee HM, Dubois J, Qiu D, Hitti J, Wolf YI, Tatusov RL, Sabathe F, Doucette-Stamm L, Soucaille P, Daly MJ, Bennett GN, Koonin EV, Smith DR, GTC Sequencing Center Production, Finishing, and Bioinformatics Teams (2001). Genome sequence and comparative analysis of the solvent-producing bacterium *Clostridium acetobutylicum*.. J Bacteriol.

[B14] Lyristis M, Boynton ZL, Petersen D, Kan Z, Bennett GN, Rudolph FB (2000). Cloning, sequencing, and characterization of the gene encoding flagellin, *flaC*, and the post-translational modification of flagellin, FlaC, from *Clostridium acetobutylicum *ATCC824.. Anaerobe.

[B15] Welch M, Oosawa K, Aizawa SI, Eisenbach M (1994). Effects of phosphorylation, Mg2+, and conformation of the chemotaxis protein CheY on its binding to the flagellar switch protein FliM.. Biochemistry.

[B16] Baer SH, Blaschek HP, Smith TL (1987). Effect of butanol challenge and temperature on lipid composition and membrane fluidity of butanol-tolerant *Clostridium acetobutylicum*.. Appl Environ Microbiol.

[B17] Lepage C, Fayolle F, Hermann M, Vandecasteele J-P (1987). Changes in membrane lipid composition of *Clostridium acetobutylicum* during acetone-butanol fermentation: effects of solvents, growth temperature and pH.. J Gen Microbiol.

[B18] Vollherbst-Schneck K, Sands JA, Montenecourt BS (1984). Effect of butanol on lipid composition and fluidity of *Clostridium acetobutylicum *ATCC 824.. Appl Environ Microbiol.

[B19] Zhao Y, Hindorff LA, Chuang A, Monroe-Augustus M, Lyristis M, Harrison ML, Rudolph FB, Bennett GN (2003). Expression of a cloned cyclopropane fatty acid synthase gene reduces solvent formation in *Clostridium acetobutylicum *ATCC 824.. Appl Environ Microbiol.

[B20] Peguin S, Soucaille P (1995). Modulation of carbon and electron flow in *Clostridium acetobutylicum *by iron limitation and methyl viologen addition.. Appl Environ Microbiol.

[B21] Jones DT, Woods DR (1986). Acetone-butanol fermentation revisited.. Microbiol Rev.

[B22] Cornillot E, Nair RV, Papoutsakis ET, Soucaille P (1997). The genes for butanol and acetone formation in *Clostridium acetobutylicum *ATCC 824 reside on a large plasmid whose loss leads to degeneration of the strain.. J Bacteriol.

[B23] Schaffer S, Isci N, Zickner B, Dürre P (2002). Changes in protein synthesis and identification of proteins specifically induced during solventogenesis in *Clostridium acetobutylicum*.. Electrophoresis.

[B24] Mansilla MC, Cybulski LE, Albanesi D, de Mendoza D (2004). Control of membrane lipid fluidity by molecular thermosensors.. J Bacteriol.

[B25] Kaan T, Homuth G, Mader U, Bandow J, Schweder T (2002). Genome-wide transcriptional profiling of the *Bacillus subtilis *cold-shock response.. Microbiology.

[B26] Johnston NC, Goldfine H (1983). Lipid composition in the classification of the butyric acid-producing clostridia.. J Gen Microbiol.

[B27] Burbulys D, Trach KA, Hoch JA (1991). Initiation of sporulation in *B. subtilis *is controlled by a multicomponent phosphorelay.. Cell.

[B28] Stragier P, Losick R (1996). Molecular genetics of sporulation in *Bacillus subtilis*.. Annu Rev Genet.

[B29] Harris LM, Welker NE, Papoutsakis ET (2002). Northern, morphological, and fermentation analysis of spo0A inactivation and overexpression in *Clostridium acetobutylicum *ATCC 824.. J Bacteriol.

[B30] Molle V, Fujita M, Jensen ST, Eichenberger P, Gonzalez-Pastor JE, Liu JS, Losick R (2003). The Spo0A regulon of *Bacillus subtilis*.. Mol Microbiol.

[B31] Cervin MA, Lewis RJ, Brannigan JA, Spiegelman GB (1998). The *Bacillus subtilis *regulator SinR inhibits *spoIIG *promoter transcription *in vitro *without displacing RNA polymerase.. Nucleic Acids Res.

[B32] Mandic-Mulec I, Doukhan L, Smith I (1995). The *Bacillus subtilis *SinR protein is a repressor of the key sporulation gene *spo0A*.. J Bacteriol.

[B33] Scotcher MC, Rudolph FB, Bennett GN (2005). Expression of *abrB310 *and *sinR*, and effects of decreased *abrB310 *expression on the transition from acidogenesis to solventogenesis, in *Clostridium acetobutylicum *ATCC 824.. Appl Environ Microbiol.

[B34] Perego M, Spiegelman GB, Hoch JA (1988). Structure of the gene for the transition state regulator, *abrB*: regulator synthesis is controlled by the *spo0A *sporulation gene in *Bacillus subtilis*.. Mol Microbiol.

[B35] Chary VK, Meloni M, Hilbert DW, Piggot PJ (2005). Control of the expression and compartmentalization of (sigma)G activity during sporulation of *Bacillus subtilis *by regulators of (sigma)F and (sigma)E.. J Bacteriol.

[B36] Sun DX, Cabrera-Martinez RM, Setlow P (1991). Control of transcription of the *Bacillus subtilis spoIIIG *gene, which codes for the forespore-specific transcription factor sigma G.. J Bacteriol.

[B37] Paredes CJ, Rigoutsos I, Papoutsakis ET (2004). Transcriptional organization of the *Clostridium acetobutylicum *genome.. Nucleic Acids Res.

[B38] Kroos L, Kunkel B, Losick R (1989). Switch protein alters specificity of RNA polymerase containing a compartment-specific sigma factor.. Science.

[B39] Stragier P, Kunkel B, Kroos L, Losick R (1989). Chromosomal rearrangement generating a composite gene for a developmental transcription factor.. Science.

[B40] Sauer U, Treuner A, Buchholz M, Santangelo JD, Dürre P (1994). Sporulation and primary sigma factor homologous genes in *Clostridium acetobutylicum*.. J Bacteriol.

[B41] Santangelo JD, Kuhn A, Treuner-Lange A, Dürre P (1998). Sporulation and time course expression of sigma-factor homologous genes in *Clostridium acetobutylicum*.. FEMS Microbiol Lett.

[B42] Tatti KM, Jones CH, Moran CP (1991). Genetic evidence for interaction of sigma E with the *spoIIID *promoter in *Bacillus subtilis*.. J Bacteriol.

[B43] Piggot PJ, Hilbert DW (2004). Sporulation of *Bacillus subtilis*.. Curr Opin Microbiol.

[B44] Wörner K, Szurmant H, Chiang C, Hoch JA (2006). Phosphorylation and functional analysis of the sporulation initiation factor Spo0A from *Clostridium botulinum*.. Mol Microbiol.

[B45] Jiang M, Shao W, Perego M, Hoch JA (2000). Multiple histidine kinases regulate entry into stationary phase and sporulation in *Bacillus subtilis*.. Mol Microbiol.

[B46] Dartois V, Djavakhishvili T, Hoch JA (1996). Identification of a membrane protein involved in activation of the KinB pathway to sporulation in *Bacillus subtilis*.. J Bacteriol.

[B47] LeDeaux JR, Grossman AD (1995). Isolation and characterization of *kinC*, a gene that encodes a sensor kinase homologous to the sporulation sensor kinases KinA and KinB in *Bacillus subtilis*.. J Bacteriol.

[B48] Alsaker KV, Paredes CJ, Papoutsakis ET (2005). Design, optimization and validation of genomic DNA microarrays for examining the *Clostridium acetobutylicum *transcriptome.. Biotechnol Bioprocess Eng.

[B49] Scotcher MC, Bennett GN (2005). SpoIIE regulates sporulation but does not directly affect solventogenesis in *Clostridium acetobutylicum *ATCC 824.. J Bacteriol.

[B50] Green EM, Boynton ZL, Harris LM, Rudolph FB, Papoutsakis ET, Bennett GN (1996). Genetic manipulation of acid formation pathways by gene inactivation in *Clostridium acetobutylicum *ATCC 824.. Microbiology.

[B51] Huang IH, Waters M, Grau RR, Sarker MR (2004). Disruption of the gene (*spo0A*) encoding sporulation transcription factor blocks endospore formation and enterotoxin production in enterotoxigenic *Clostridium perfringens *type A.. FEMS Microbiol Lett.

[B52] Raju D, Waters M, Setlow P, Sarker MR (2006). Investigating the role of small, acid-soluble spore proteins (SASPs) in the resistance of *Clostridium perfringens *spores to heat.. BMC Microbiol.

[B53] Sarker MR, Carman RJ, McClane BA (1999). Inactivation of the gene (*cpe*) encoding *Clostridium perfringens *enterotoxin eliminates the ability of two cpe-positive *C. perfringens *type A human gastrointestinal disease isolates to affect rabbit ileal loops.. Mol Microbiol.

[B54] Heap JT, Pennington OJ, Cartman ST, Carter GP, Minton NP (2007). The ClosTron: a universal gene knock-out system for the genus *Clostridium*.. J Microbiol Methods.

[B55] Desai RP, Papoutsakis ET (1999). Antisense RNA strategies for metabolic engineering of *Clostridium acetobutylicum*.. Appl Environ Microbiol.

[B56] Tummala SB, Welker NE, Papoutsakis ET (2003). Design of antisense RNA constructs for downregulation of the acetone formation pathway of *Clostridium acetobutylicum*.. J Bacteriol.

[B57] Perret S, Maamar H, Belaich JP, Tardif C (2004). Use of antisense RNA to modify the composition of cellulosomes produced by *Clostridium cellulolyticum*.. Mol Microbiol.

[B58] Raju D, Setlow P, Sarker MR (2007). Antisense-RNA-mediated decreased synthesis of small, acid-soluble spore proteins leads to decreased resistance of *Clostridium perfringens *spores to moist heat and UV radiation.. Appl Environ Microbiol.

[B59] Tummala SB, Junne SG, Papoutsakis ET (2003). Antisense RNA downregulation of coenzyme A transferase combined with alcohol-aldehyde dehydrogenase overexpression leads to predominantly alcohologenic *Clostridium acetobutylicum *fermentations.. J Bacteriol.

[B60] Tomas CA, Welker NE, Papoutsakis ET (2003). Overexpression of *groESL *in *Clostridium acetobutylicum *results in increased solvent production and tolerance, prolonged metabolism, and changes in the cell's transcriptional program.. Appl Environ Microbiol.

[B61] Asai K, Ishiwata K, Matsuzaki K, Sadaie Y (2008). A viable *Bacillus subtilis *strain without functional extracytoplasmic function sigma genes.. J Bacteriol.

[B62] Mascher T, Hachmann AB, Helmann JD (2007). Regulatory overlap and functional redundancy among *Bacillus subtilis *extracytoplasmic function sigma factors.. J Bacteriol.

[B63] Paredes CJ, Senger RS, Spath IS, Borden JR, Sillers R, Papoutsakis ET (2007). A general framework for designing and validating oligomer-based DNA microarrays and its application to *Clostridium acetobutylicum*.. Appl Environ Microbiol.

[B64] Saeed AI, Sharov V, White J, Li J, Liang W, Bhagabati N, Braisted J, Klapa M, Currier T, Thiagarajan M, Sturn A, Snuffin M, Rezantsev A, Popov D, Ryltsov A, Kostukovich E, Borisovsky I, Liu Z, Vinsavich A, Trush V, Quackenbush J (2003). TM4: a free, open-source system for microarray data management and analysis.. Biotechniques.

[B65] Reynolds ES (1963). The use of lead citrate at high pH as an electron-opaque stain in electron microscopy.. J Cell Biol.

[B66] Frey M (2002). Hydrogenases: hydrogen-activating enzymes.. Chembiochem.

[B67] Gorwa MF, Croux C, Soucaille P (1996). Molecular characterization and transcriptional analysis of the putative hydrogenase gene of *Clostridium acetobutylicum *ATCC 824.. J Bacteriol.

[B68] Moir A, Corfe BM, Behravan J (2002). Spore germination.. Cell Mol Life Sci.

[B69] Igarashi T, Setlow P (2006). Transcription of the *Bacillus subtilis gerK *operon, which encodes a spore germinant receptor, and comparison with that of operons encoding other germinant receptors.. J Bacteriol.

[B70] Dürre P, Hollergschwandner C (2004). Initiation of endospore formation in *Clostridium acetobutylicum*.. Anaerobe.

[B71] Makino S, Moriyama R (2002). Hydrolysis of cortex peptidoglycan during bacterial spore germination.. Med Sci Monit.

[B72] Ishikawa S, Yamane K, Sekiguchi J (1998). Regulation and characterization of a newly deduced cell wall hydrolase gene (*cwlJ*) which affects germination of *Bacillus subtilis *spores.. J Bacteriol.

[B73] Kodama T, Takamatsu H, Asai K, Kobayashi K, Ogasawara N, Watabe K (1999). The *Bacillus subtilis yaaH *gene is transcribed by SigE RNA polymerase during sporulation, and its product is involved in germination of spores.. J Bacteriol.

[B74] Moriyama R, Fukuoka H, Miyata S, Kudoh S, Hattori A, Kozuka S, Yasuda Y, Tochikubo K, Makino S (1999). Expression of a germination-specific amidase, SleB, of bacilli in the forespore compartment of sporulating cells and its localization on the exterior side of the cortex in dormant spores.. J Bacteriol.

[B75] Setlow P (1994). Mechanisms which contribute to the long-term survival of spores of *Bacillus *species.. Soc Appl Bacteriol Symp Ser.

[B76] Bourne N, FitzJames PC, Aronson AI (1991). Structural and germination defects of *Bacillus subtilis *spores with altered contents of a spore coat protein.. J Bacteriol.

[B77] Roels S, Driks A, Losick R (1992). Characterization of *spoIVA*, a sporulation gene involved in coat morphogenesis in *Bacillus subtilis*.. J Bacteriol.

[B78] Takamatsu H, Imamura A, Kodama T, Asai K, Ogasawara N, Watabe K (2000). The *yabG *gene of *Bacillus subtilis *encodes a sporulation specific protease which is involved in the processing of several spore coat proteins.. FEMS Microbiol Lett.

[B79] Takamatsu H, Watabe K (2002). Assembly and genetics of spore protective structures.. Cell Mol Life Sci.

[B80] Driks A, Sonenshein AL, Hoch JA, Losick R (2002). Proteins of the spore core and coat.. Bacillus subtilis and its Closest Relatives: From Genes to Cells.

[B81] Britton RA, Eichenberger P, Gonzalez-Pastor JE, Fawcett P, Monson R, Losick R, Grossman AD (2002). Genome-wide analysis of the stationary-phase sigma factor (sigma-H) regulon of *Bacillus subtilis*.. J Bacteriol.

[B82] Wang ST, Setlow B, Conlon EM, Lyon JL, Imamura D, Sato T, Setlow P, Losick R, Eichenberger P (2006). The forespore line of gene expression in *Bacillus subtilis*.. J Mol Biol.

[B83] Eichenberger P, Fujita M, Jensen ST, Conlon EM, Rudner DZ, Wang ST, Ferguson C, Haga K, Sato T, Liu JS, Losick R (2004). The program of gene transcription for a single differentiating cell type during sporulation in *Bacillus subtilis*.. PLoS Biol.

