# Supplementary material for: The transcriptional program underlying the physiology of clostridial sporulation
Source: Genome Biol. 2008 Jul 16;9(7):R114. doi: 10.1186/gb-2008-9-7-r114 (PMC2530871; doi:10.1186/gb-2008-9-7-r114)
Supplement: Additional data file 1 — Comparison of the present microarray study to an earlier microarray study that examined the early sporulation of C. acetobutylicum. [file gb-2008-9-7-r114-S1.pdf]

Validation of Microarray Data

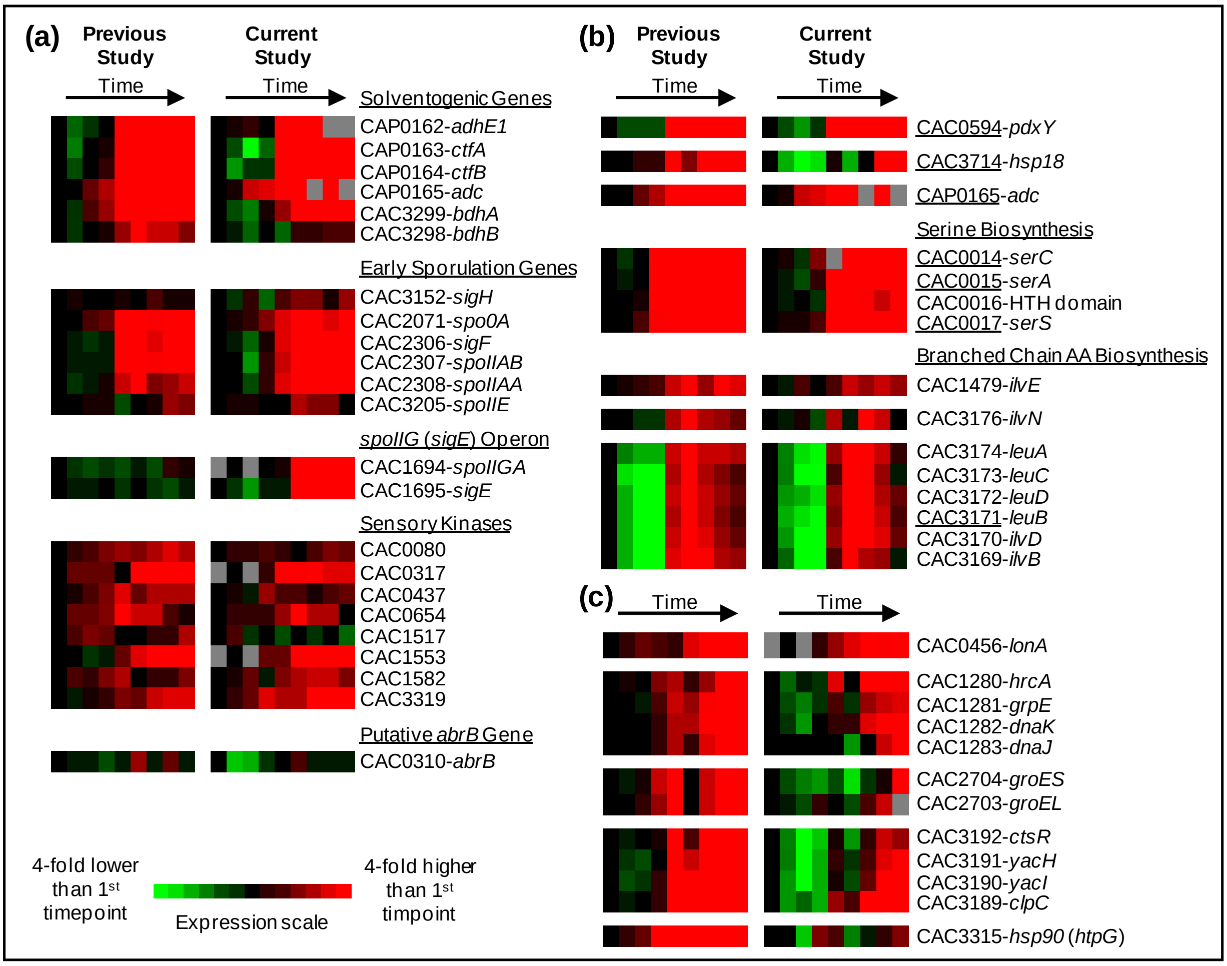

Figure S1 - Comparison of expression profiles of specific functional groups from two studies

Expression values from timepoints with similar OD_{600} values are compared and presented as ratios compared to the first expressed timepoint with genes having a higher expression shown as red and those with a lower expression as green. Saturated expression level: 4-fold difference (see scale). Gray squares indicate timepoints at which the intensity did not exceed the threshold value. (a) Genes related to solventogenesis and sporulation. (b) Genes whose protein products were identified by Schaffer et al [23] to be differentially expressed during solventogenesis. Underlined

genes were specifically identified by Schaffer et al [23], while all others are related genes. (c) Genes encoding for heat shock proteins.

Validation of Microarray Data

The microarray results from this study were validated by quantitative (Q) RT-PCR using RNA samples from a biological replicate culture, and by a detailed comparison to an earlier microarray study that examined the early sporulation of *C. acetobutylicum* [7].

A selected set of sporulation related genes were examined using Q-RT-PCR. Samples were taken every six hours starting at hour 6 and continuing until hour 48. The early sporulation related genes *abrB* and *sinR* all peak in expression during hour 12, as does *spo0A* (Figure 2). Those genes do however show a more pronounced expression than is evident in the microarray results. The sporulation-related sigma factors *sigF*, *sigE*, and *sigG* all show the bimodal expression profile evident during the microarray analysis. *spoIIE*, *spoIIID*, and *spoIIIAA*, all show very similar patterns of induction compared to the microarray results. Genes whose expression levels did not meet the expression cutoff in the microarray analysis were also used for this comparison, and those results compare favorably; thus further demonstrating the exceptional sensitivity and accuracy (supported by an independent set of experiments [63]; data not shown) of this microarray platform.

For the comparison of the data to a previous limited study using a different microarray platform [7], the expression profiles for all experiments were expressed as a ratio compared to the first timepoint to remove the influence of different reference RNA pools in the microarray studies. If data were missing for the first timepoint, the second timepoint was used for comparisons. Only

timepoints with similar OD_{600} values were compared. The data between the two studies agree well overall. The profiles of the solventogenic genes, *adhE1-ctfA-ctfB*, *adc*, *bdhA*, and *bdhB*, are in close agreement with the two sets of data (Figure S1a). The early sporulation genes, *sigH*, *spo0A*, *sigF*, *spoIIAB*, *spoIIAA*, and *spoIIE*, also agree very well between the two data sets. However, the expression profiles for the *spoIIG* (*sigE*) operon display dramatic differences. The profile from the older platform shows no significant upregulation of the operon, whereas the profile from the new platform has a dramatic upregulation shortly after expression of *spo0A*, in accordance with the proposed regulatory model of *sigE* [41]. It is also confirmed by the Q-RT-PCR analysis discussed above. The profiles from different sensory kinases and *abrB* agree well, with the possible exception of CAC1517. Using two-dimensional gel electrophoresis, Schaffer et al [23] identified proteins induced during solventogenesis. The transcription profiles for those genes all exhibit an upregulation at the onset of solventogenesis (Figure S1b). The previous microarray study also identified the induction of genes involved in stress response, and the same induction is observed with the current microarray platform although it seems to slightly be delayed compared to the earlier study (Figure S1c).
