# Supplementary material for: The transcriptional program underlying the physiology of clostridial sporulation
Source: Genome Biol. 2008 Jul 16;9(7):R114. doi: 10.1186/gb-2008-9-7-r114 (PMC2530871; doi:10.1186/gb-2008-9-7-r114)
Supplement: Additional data file 3 — Transcriptional profiles, in terms of both intensity and differential expression, of specific gene clusters. [file gb-2008-9-7-r114-S3.pdf]

Transcriptional profiles, in terms of both intensity and differential expression, of specific gene clusters

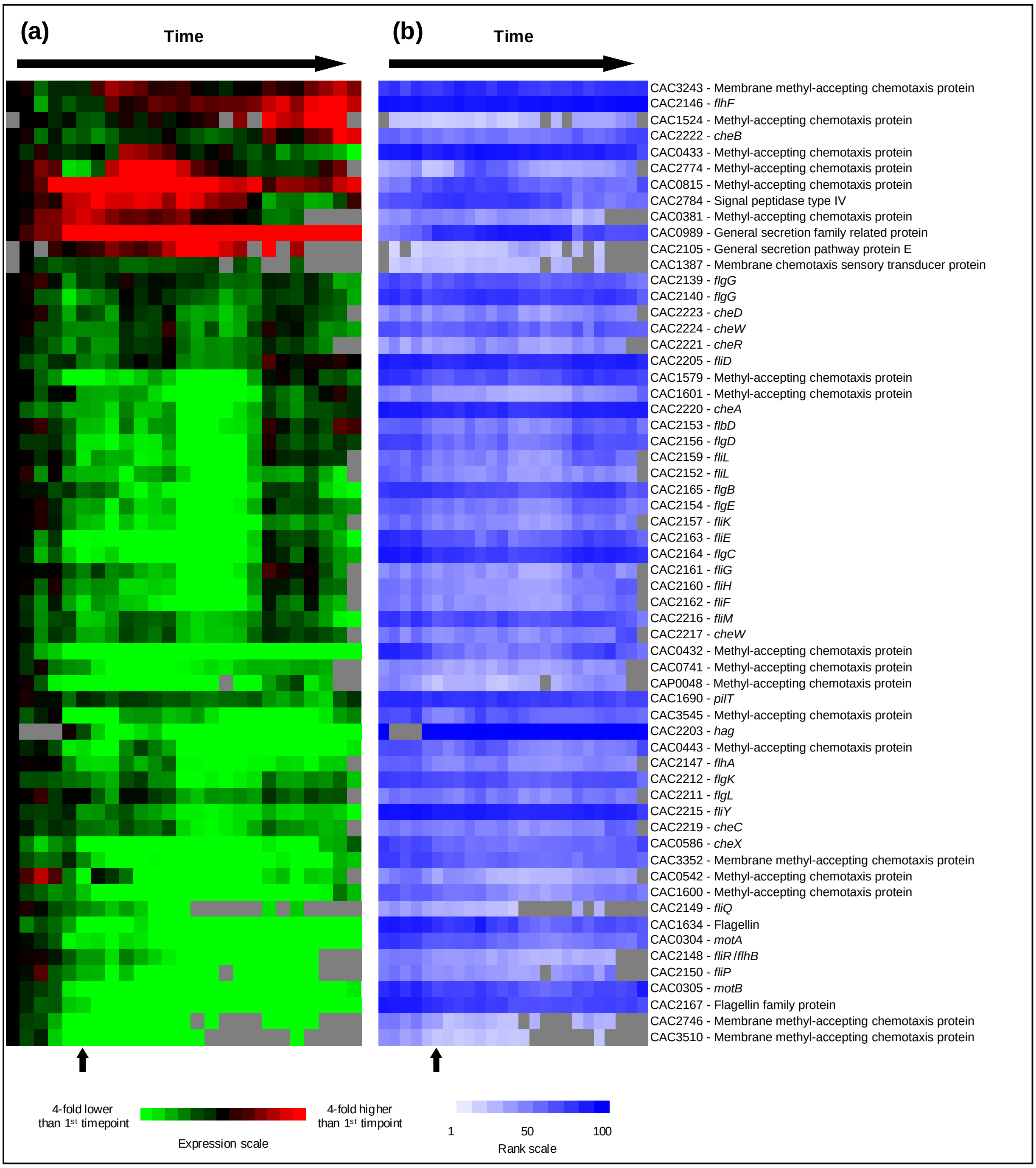

Figure S2 - Expression profiles of genes related to cell motility and secretion (COG class N)

Genes with more than eight consecutively expressed timepoints were hierarchically clustered using the Pearson correlation. Because of the cutoff of eight timepoints, thirty-three genes within this COG class were not clustered. Arrows denote the onset of transitional phase. Gray squares indicate timepoints at which the intensity did not exceed the threshold value. (a) Expression values are presented as ratios compared to the first expressed timepoint, with genes having a higher expression shown as red and those with a lower expression as green. Saturated expression level: 4-fold difference (see scale). (b) Ranked expression intensity values for each gene. Ranks run from 100 to 1 with 100 being blue and 1 being white (see scale).

Cell motility and secretion

Stationary phase cells are visibly less motile, so a general downregulation of genes related to cell motility and secretion (COG class N) is to be expected and has been reported earlier by us [1]. The majority of known motility and chemotaxis genes fall within two gene clusters on the chromosome. On the first cluster are a large number of genes related to flagellar machinery (*flgBCDEG* and *fliEFGHJKLPQZ*), while on the second are many of the known chemotaxis genes (*cheABCDRWY*). Most of these genes fall within the middle of the plot (from CAC2139-*flgG* to CAC2217-*cheW*) and display a bimodal expression pattern. As expected, these genes are downregulated just before the onset of stationay phase, but many are again upregulated towards the end of the timecourse, around hour 38. This late expression is consistent with the microscopy, which observed highly motile vegetative cells late in the timecourse. CAC2139-40 (*flgG*), CAC2221,23-24 (*cheRDWI*), and CAC2205 (*fliD*) are also upregulated mid-stationary phase for about 6 hours. Four other less-characterized chemotaxis proteins (CAC0433,

CAC2774, CAC0815, and CAC0381) are upregulated during stationary phase along with *flhF* (CAC2146), two general secretion proteins (CAC0989 and CAC2105) and a signal peptidase (CAC2784). The importance of these genes is not known.

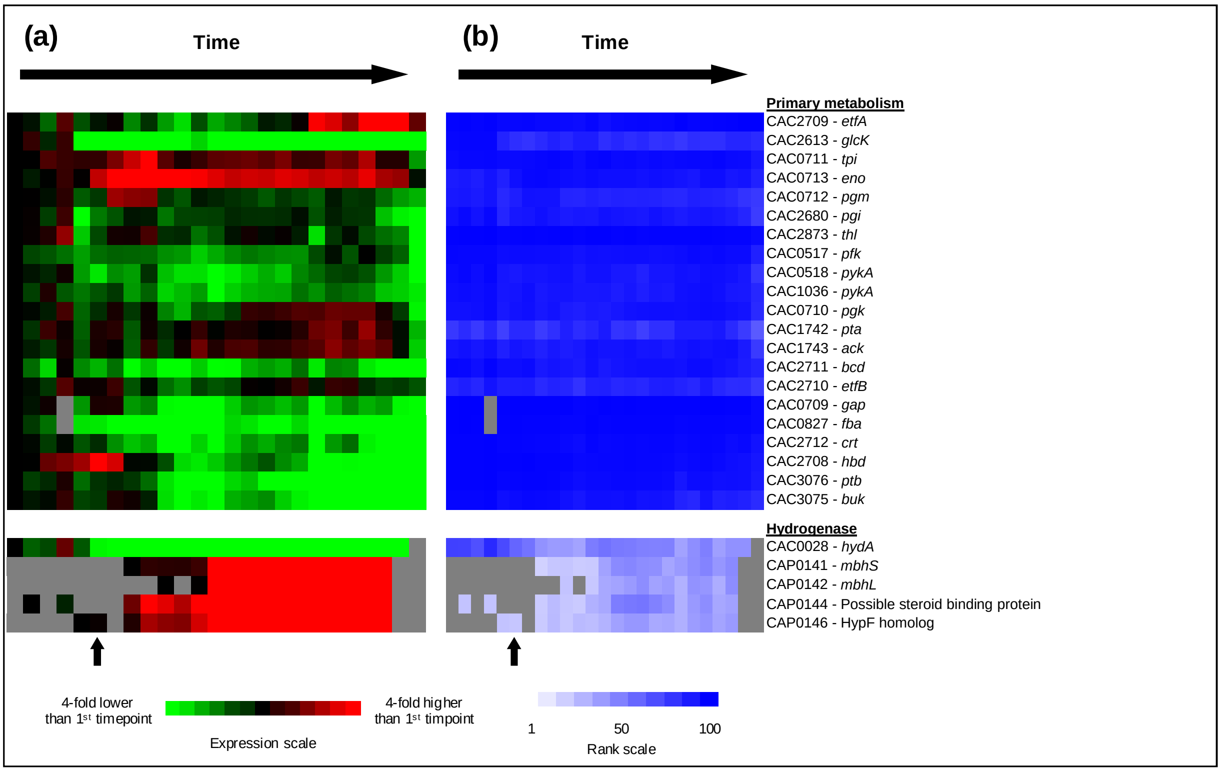

Figure S3 - Expression profiles of primary metabolism genes and hydrogenases of *C. acetobutylicum*

Genes were hierarchically clustered using the Pearson correlation. Arrows denote the onset of transitional phase. Gray squares indicate timepoints at which the intensity did not exceed the threshold value. (a) Expression values are presented as ratios compared to the first expressed timepoint, with genes having a higher expression shown as red and those with a lower expression as green. Saturated expression level: 4-fold difference (see scale). (b) Ranked expression intensity values for each gene. Ranks run from 100 to 1 with 100 being blue and 1 being white (see scale).

Primary metabolism enzymes and hydrogenases

The enzymes involved in glycolysis display varying expression patterns but are all highly expressed throughout the timecourse (Figure S3). The glucokinase *glcK*, the 6-

phosphofructoskinase (*pfk*), two pyruvate kinases (*pykA*, CAC0518 and CAC1036), the glyceraldehydes 3-phophate dehydrogenase (*gap*), and the fructose-biphosphate aldolase (*fba*) were all downregulated early in the timecourse, by hour 10, while the triosephosphate isomerase (*tpi*) and the enolase (*eno*) were highly expressed throughout stationary phase. The remaining enzymes, the phosphoglycerate mutase (*pgm*), the glucose-6-phosphate isomerase (*pgi*), and the phosphoglycerate kinase (*pgk*) all displayed fairly flat profiles, though *pgk* was slightly upregulated during late stationary phase. The enzymes leading to acetate formation, phosphate acetyltransferase (*pta*) and acetate kinase (*ack*), also had a fairly flat expression profile with a slight upregulation late stationary phase. In contrast, the enzymes leading to butyrate formation, phosphate butyryltransferase (*ptb*) and butyrate kinase (*buk*), were downregulated during early stationary phase. The enzymes which convert acetyl-CoA to butyryl-CoA, required for both butyrate and butanol formation, exhibited a variable expression pattern. Expression of the thiolase (*thl*) was fairly flat but did drop slightly during late exponential phase and rose again during early stationary phase. The crotonase (*crt*) was downregulated throughout stationary phase as was the 3-hydroxybutyryl-CoA dehydrogenase (*hbd*) after an upregulation during transitional phase. The butyryl-CoA dehydrogenase (*bcd*) was downregulated throughout late exponential phase and stationary phase, while the electron transfer flavoprotein α and β subunits (*etfA*/*B*), required for BCD activity, were downregulated early stationary phase and upregulated again late stationary phase.

On the *C. acetobutylicum* genome, two classes of hydrogenases are found: the iron only (FeFe) and nickel-iron (NiFe). *hydA* (CAC0028) encodes for the iron only hydrogenase while *mbhS*, small subunit, and *mbhL*, large subunit, on the operon (CAP0141-6) encodes for the nickel-iron

hydrogenase. Typically, FeFe hydrogenases produce hydrogen, while NiFe hydrogenases consume and oxidize hydrogen [66]. It is known that during acidogenesis, molecular hydrogen is produced in order to regenerate ferredoxin, which in turn reduces NADH produced during glycoylsis. The hydrogenase *hydA*, an iron only hydrogenase, is associated with this reaction [67]. As the culture switches from acidogenesis to solventogenesis, the NADH pool is consumed instead by solvent generation, and thus the hydrogenase is no longer needed and is downregulated [7,67]. *hydA* expression in our study was downregulated just before the onset of stationary phase and remained downregulated, consistent with the previous reports. In contrast, the nickel-iron hydrogenase, *mbhS* and *mbhL*, was sharply upregulated during mid-stationary phase, hour 26, and remained upregulated until late stationary phase, hour 54. Expressed on the same operon are CAP0144, a possible steroid binding protein, and CAP0146, a HypF homolog involved in hydrogenase maturation. Being a NiFe hydrogenase, the enzyme encoded by *mbhS*/*L* may reverse the previous reaction in order to produce more NAD(P)H for solvent formation.

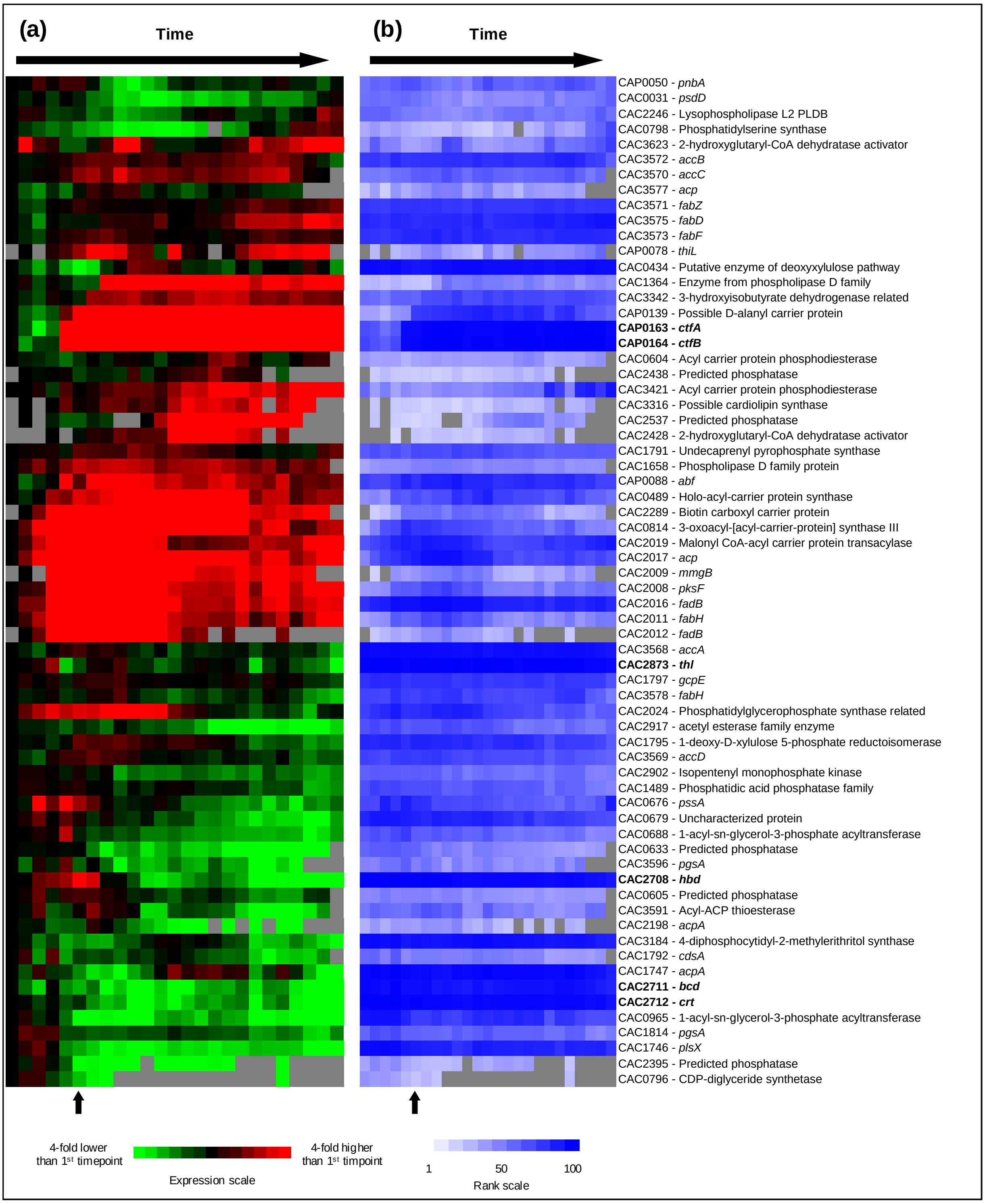

Figure S4 - Expression profiles of genes related to lipid metabolism (COG class I)

Genes with more than eight consecutively expressed timepoints were hierarchically clustered using the Pearson correlation. Because of the cutoff of eight timepoints, three genes within this COG class were not clustered. Several acidogenic and solventogenic enzymes are classified within this COG class and are denoted in bold. Arrows denote the onset of transitional phase. Gray squares indicate timepoints at which the intensity did not exceed the threshold value. (a) Expression values are presented as ratios compared to the first expressed timepoint, with genes having a higher expression shown as red and those with a lower expression as green. Saturated expression level: 4-fold difference (see scale). (b) Ranked expression intensity values for each gene. Ranks run from 100 to 1 with 100 being blue and 1 being white (see scale).

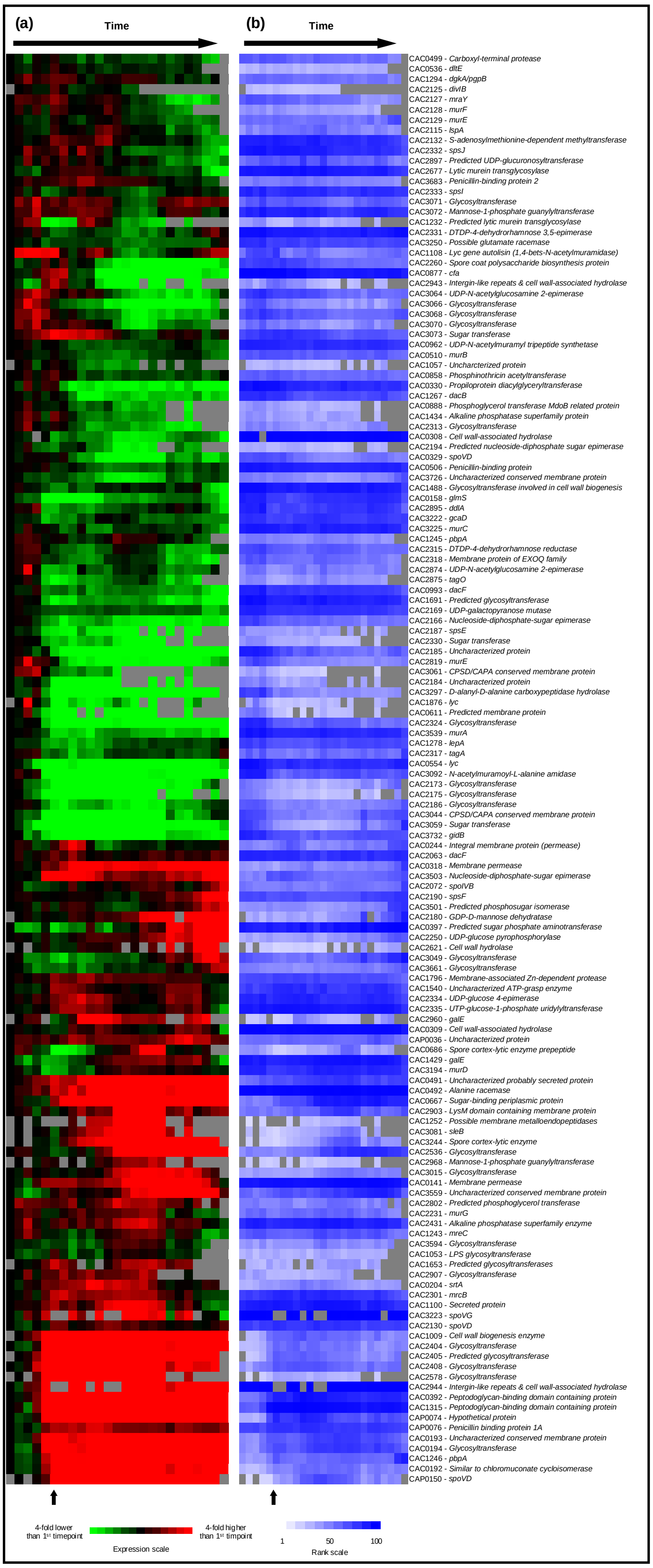

(a)
Time
(b)
Time
CAC0499 - Carboxyl-terminal protease
CAC0536 - dltE
CAC1294 - dgkA/pgpB
CAC2125 - divIB
CAC2127 - mraY
CAC2128 - murF
CAC2129 - murE
CAC2115 - lspA
CAC2132 - S-adenosylmethionine-dependent methyltransferase
CAC2332 - spsJ
CAC2897 - Predicted UDP-glucuronosyltransferase
CAC2677 - Lytic murein transglycosylase
CAC3683 - Penicillin-binding protein 2
CAC2333 - spsI
CAC3071 - Glycosyltransferase
CAC3072 - Mannose-1-phosphate guanylyltransferase
CAC1232 - Predicted lytic murein transglycosylase
CAC2331 - DTDP-4-dehydrorhamnose 3,5-epimerase
CAC3250 - Possible glutamate racemase
CAC1108 - Lyc gene autolisin (1,4-bets-N-acetylmuramidase)
CAC2260 - Spore coat polysaccharide biosynthesis protein
CAC0877 - cfa
CAC2943 - Intergin-like repeats & cell wall-associated hydrolase
CAC3064 - UDP-N-acetylglucosamine 2-epimerase
CAC3066 - Glycosyltransferase
CAC3068 - Glycosyltransferase
CAC3070 - Glycosyltransferase
CAC3073 - Sugar transferase
CAC0962 - UDP-N-acetylmuramyl tripeptide synthetase
CAC0510 - murB
CAC1057 - Uncharcterized protein
CAC0858 - Phosphinothricin acetyltransferase
CAC0330 - Propiloprotein diacylglyceryltransferase
CAC1267 - dacB
CAC0888 - Phosphoglycerol transferase MdoB related protein
CAC1434 - Alkaline phosphatase superfamily protein
CAC2313 - Glycosyltransferase
CAC0308 - Cell wall-associated hydrolase
CAC2194 - Predicted nucleoside-diphosphate sugar epimerase
CAC0329 - spoVD
CAC0506 - Penicillin-binding protein
CAC3726 - Uncharacterized conserved membrane protein
CAC1488 - Glycosyltransferase involved in cell wall biogenesis
CAC0158 - glmS
CAC2895 - ddlA
CAC3222 - gcaD
CAC3225 - murC
CAC1245 - pbpA
CAC2315 - DTDP-4-dehydrorhamnose reductase
CAC2318 - Membrane protein of EXOQ family
CAC2874 - UDP-N-acetylglucosamine 2-epimerase
CAC2875 - tagO
CAC0993 - dacF
CAC1691 - Predicted glycosyltransferase
CAC2169 - UDP-galactopyranose mutase
CAC2166 - Nucleoside-diphosphate-sugar epimerase
CAC2187 - spsE
CAC2330 - Sugar transferase
CAC2185 - Uncharacterized protein
CAC2819 - murE
CAC3061 - CPSD/CAPA conserved membrane protein
CAC2184 - Uncharacterized protein
CAC3297 - D-alanyl-D-alanine carboxypeptidase hydrolase
CAC1876 - lyc
CAC0611 - Predicted membrane protein
CAC2324 - Glycosyltransferase
CAC3539 - murA
CAC1278 - lepA
CAC2317 - tagA
CAC0554 - lyc
CAC3092 - N-acetylmuramoyl-L-alanine amidase
CAC2173 - Glycosyltransferase
CAC2175 - Glycosyltransferase
CAC2186 - Glycosyltransferase
CAC3044 - CPSD/CAPA conserved membrane protein
CAC3059 - Sugar transferase
CAC3732 - gidB
CAC0244 - Integral membrane protein (permease)
CAC2063 - dacF
CAC0318 - Membrane permease
CAC3503 - Nucleoside-diphosphate-sugar epimerase
CAC2072 - spoIVB
CAC2190 - spsF
CAC3501 - Predicted phosphosugar isomerase
CAC2180 - GDP-D-mannose dehydratase
CAC0397 - Predicted sugar phosphate aminotransferase
CAC2250 - UDP-glucose pyrophosphorylase
CAC2621 - Cell wall hydrolase
CAC3049 - Glycosyltransferase
CAC3661 - Glycosyltransferase
CAC1796 - Membrane-associated Zn-dependent protease
CAC1540 - Uncharacterized ATP-grasp enzyme
CAC2334 - UDP-glucose 4-epimerase
CAC2335 - UTP-glucose-1-phosphate uridylyltransferase
CAC2960 - galE
CAC0309 - Cell wall-associated hydrolase
CAP0036 - Uncharacterized protein
CAC0686 - Spore cortex-lytic enzyme prepeptide
CAC1429 - galE
CAC3194 - murD
CAC0491 - Uncharacterized probably secreted protein
CAC0492 - Alanine racemase
CAC0667 - Sugar-binding periplasmic protein
CAC2903 - LysM domain containing membrane protein
CAC1252 - Possible membrane metalloendopeptidases
CAC3081 - sleB
CAC3244 - Spore cortex-lytic enzyme
CAC2536 - Glycosyltransferase
CAC2968 - Mannose-1-phosphate guanylyltransferase
CAC3015 - Glycosyltransferase
CAC0141 - Membrane permease
CAC3559 - Uncharacterized conserved membrane protein
CAC2802 - Predicted phosphoglycerol transferase
CAC2231 - murG
CAC2431 - Alkaline phosphatase superfamily enzyme
CAC1243 - mreC
CAC3594 - Glycosyltransferase
CAC1053 - LPS glycosyltransferase
CAC1653 - Predicted glycosyltransferases
CAC2907 - Glycosyltransferase
CAC0204 - srtA
CAC2301 - mrcB
CAC1100 - Secreted protein
CAC3223 - spoVG
CAC2130 - spoVD
CAC1009 - Cell wall biogenesis enzyme
CAC2404 - Glycosyltransferase
CAC2405 - Predicted glycosyltransferase
CAC2408 - Glycosyltransferase
CAC2578 - Glycosyltransferase
CAC2944 - Intergin-like repeats & cell wall-associated hydrolase
CAC0392 - Peptodoglycan-binding domain containing protein
CAC1315 - Peptodoglycan-binding domain containing protein
CAP0074 - Hypothetical protein
CAP0076 - Penicillin binding protein 1A
CAC0193 - Uncharacterized conserved membrane protein
CAC0194 - Glycosyltransferase
CAC1246 - pbpA
CAC0192 - Similar to chloromuconate cycloisomerase
CAP0150 - spoVD
4-fold lower than 1st timepoint
4-fold higher than 1st timpoint
Expression scale
1
50
100
Rank scale

Figure S5 - Expression profiles of genes related to cell envelope biogenesis (COG class M)

Genes with more than eight consecutively expressed timepoints were hierarchically clustered using the Pearson correlation. Because of the cutoff of eight timepoints, forty-five genes within this COG class were not clustered. Arrows denote the onset of transitional phase. Gray squares indicate timepoints at which the intensity did not exceed the threshold value. (a) Expression values are presented as ratios compared to the first expressed timepoint, with genes having a higher expression shown as red and those with a lower expression as green. Saturated expression level: 4-fold difference (see scale). (b) Ranked expression intensity values for each gene. Ranks run from 100 to 1 with 100 being blue and 1 being white (see scale).

Changes to cell membrane composition

C. acetobutylicum is known to undergo a change in its membrane composition and fluidity in response to increasing levels of butanol [16-18]. In order to access if global changes in gene expression are associated with this change, we investigated genes related to lipid metabolism (COG class I) and cell envelope biogenesis (COG class M). Previously, analysis of cultures challenged with butanol revealed a general downregulation of the *fab* operon (fatty acid biosynthesis operon, CAC3579-68) [5], but a global investigation of a batch culture has yet to be carried out. Six genes of the *fab* operon (*accB*, *accC*, *acp, fabZ*, *fabD,* and *fabF*) clustered together near the top of Figure S4, while the remaining three genes (*accA*, *fabH*, and *accD*) fell farther down in the plot. All of these genes did not display any large temporal change over the timecourse, though the first six did have higher expression during stationary phase than late exponential phase. All genes were also highly expressed, except for *acp*. In type II fatty acid synthesis, all intermediates are covalently bound to the acyl carrier protein (ACP), making it a

critical component for fatty acid synthesis. Four genes are annotated as acyl carrier proteins (*acp*): CAC3577, in the *fab* operon, CAC2017, CAC2198, and CAC1747. CAC1747 was the most highly expressed of the *acp* genes and was upregulated mid-stationary phase, while CAC2198 was mostly downregulated during stationary phase and was lowly expressed (Figure S4). CAC2017 was highly upregulated throughout most of the timecourse and was strongly expressed (Figure S4). Other genes on the operon containing CAC2017 (CAC2019-07), the most highly upregulated *acp*, are *mmgB* (CAC2009), *pksF* (CAC2008), *fadB* (CAC2016, CAC2012), *fabH* (CAC2011), and a malonyl CoA-ACP transacylase (CAC2019). All of these genes were highly upregulated throughout transitional phase and early stationary phase and then decreased in expression for the rest of the timecourse (Figure S4). Most of the genes are associated with fatty acid synthesis, *acp*, *mmgB*, *pksF*, *fabH*, and malonyl CoA-ACP transacylase, but *fadB* is generally associated with the breakdown of fatty acids instead of synthesis. Of special interest in COG class M is *cfa* (CAC0877), the cyclopropane fatty acid synthesis gene because the ratio of cyclopropane fatty acid in the outer membrane has been shown to increase during stationary phase [18,19]. *cfa* is upregulated during transitional phase but is then downregulated throughout stationary phase (Figure S5). However, it remains highly expressed throughout the timecourse (Figure S5). Also within this COG class are a number of glycosyltransferases with different expression patterns. Three (CAC3066, CAC3068, and CAC3070) cluster close to *cfa* and are upregulated during late exponential phase (Figure S5). Four more (CAC2404, CAC2408, CAC2578, and CAC0194) cluster towards the bottom of Figure S5 and are upregulated throughout transitional phase and stationary phase. However, it is unclear the role they may play in membrane changes. Several other genes in both Figure S4 and S5 have interesting expression patterns, expressed during transitional phase, throughout stationary phase, or late stationary

phase, but the significance of these patterns is not known. Of note, several enzymes involved in both acidogenesis and solventogenesis are listed (CAP0163, CAP0162, CAC2873, CAC2708, CAC2711, and CAC2712) and indicated in bold (Figure S4). Though these enzymes fall within the COG class I, their function is known and not involved in fatty acid synthesis.

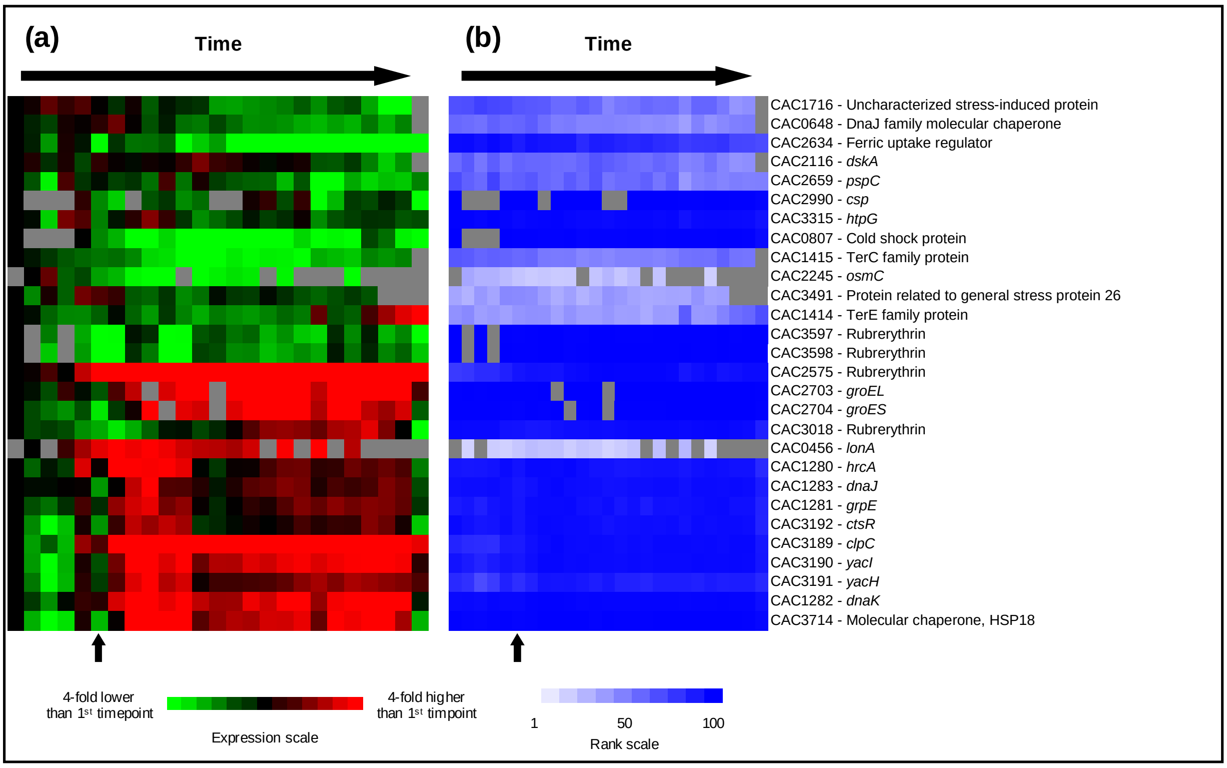

Figure S6 - Expression profiles of stress response and heat-shock proteins identified on the *C. acetobutylicum* chromosome.

Genes were hierarchically clustered using the Pearson correlation. Arrows denote the onset of transitional phase. Gray squares indicate timepoints at which the intensity did not exceed the threshold value. (a) Expression values are presented as ratios compared to the first expressed timepoint, with genes having a higher expression shown as red and those with a lower expression as green. Saturated expression level: 4-fold difference (see scale). (b) Ranked expression intensity values for each gene. Ranks run from 100 to 1 with 100 being blue and 1 being white (see scale).

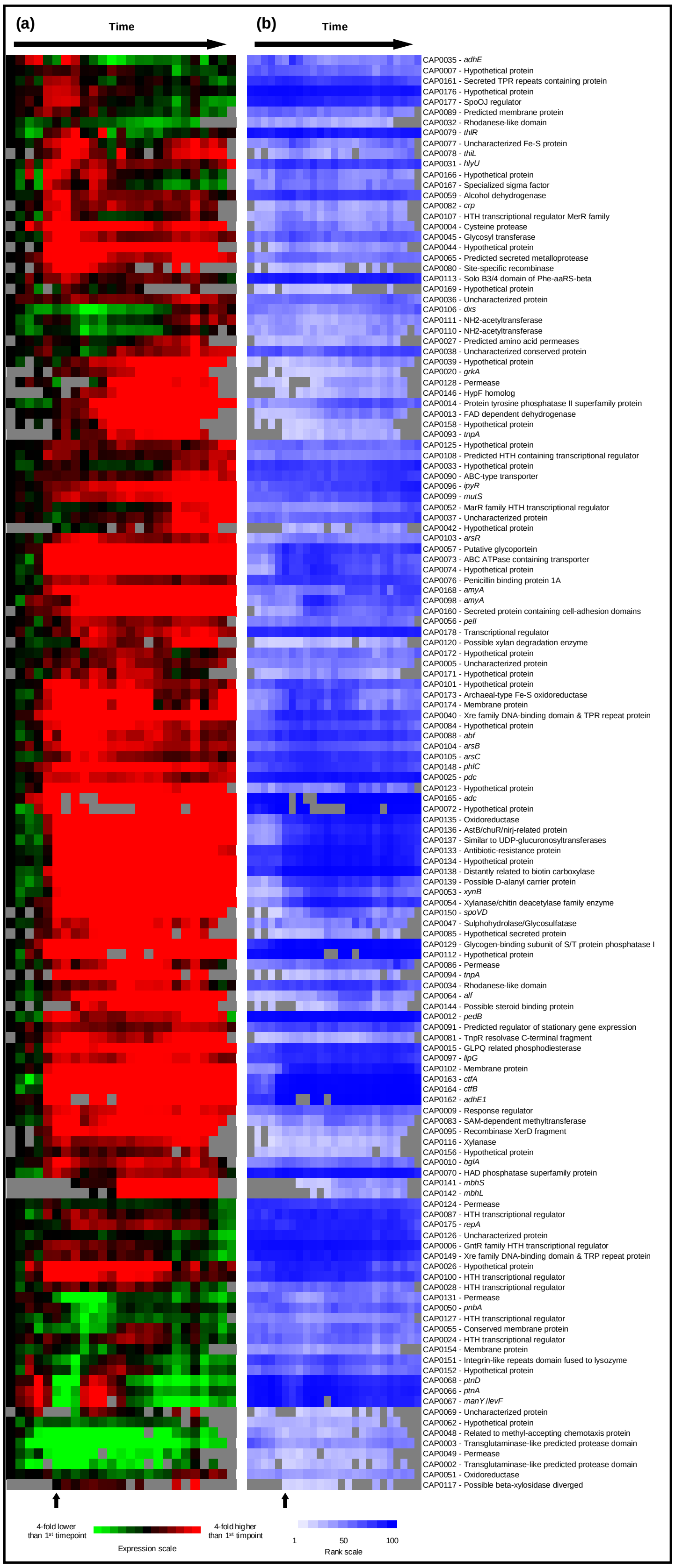
(a)
Time
(b)
Time
CAP0035 - adhE
CAP0007 - Hypothetical protein
CAP0161 - Secreted TPR repeats containing protein
CAP0176 - Hypothetical protein
CAP0177 - SpoOJ regulator
CAP0089 - Predicted membrane protein
CAP0032 - Rhodanese-like domain
CAP0079 - thlR
CAP0077 - Uncharacterized Fe-S protein
CAP0078 - thiL
CAP0031 - hlyU
CAP0166 - Hypothetical protein
CAP0167 - Specialized sigma factor
CAP0059 - Alcohol dehydrogenase
CAP0082 - crp
CAP0107 - HTH transcriptional regulator MerR family
CAP0004 - Cysteine protease
CAP0045 - Glycosyl transferase
CAP0044 - Hypothetical protein
CAP0065 - Predicted secreted metalloprotease
CAP0080 - Site-specific recombinase
CAP0113 - Solo B3/4 domain of Phe-aaRS-beta
CAP0169 - Hypothetical protein
CAP0036 - Uncharacterized protein
CAP0106 - dxs
CAP0111 - NH2-acetyltransferase
CAP0110 - NH2-acetyltransferase
CAP0027 - Predicted amino acid permeases
CAP0038 - Uncharacterized conserved protein
CAP0039 - Hypothetical protein
CAP0020 - grkA
CAP0128 - Permease
CAP0146 - HypF homolog
CAP0014 - Protein tyrosine phosphatase II superfamily protein
CAP0013 - FAD dependent dehydrogenase
CAP0158 - Hypothetical protein
CAP0093 - tnpA
CAP0125 - Hypothetical protein
CAP0108 - Predicted HTH containing transcriptional regulator
CAP0033 - Hypothetical protein
CAP0090 - ABC-type transporter
CAP0096 - ipyR
CAP0099 - mutS
CAP0052 - MarR family HTH transcriptional regulator
CAP0037 - Uncharacterized protein
CAP0042 - Hypothetical protein
CAP0103 - arsR
CAP0057 - Putative glycoportein
CAP0073 - ABC ATPase containing transporter
CAP0074 - Hypothetical protein
CAP0076 - Penicillin binding protein 1A
CAP0168 - amyA
CAP0098 - amyA
CAP0160 - Secreted protein containing cell-adhesion domains
CAP0056 - pelI
CAP0178 - Transcriptional regulator
CAP0120 - Possible xylan degradation enzyme
CAP0172 - Hypothetical protein
CAP0005 - Uncharacterized protein
CAP0171 - Hypothetical protein
CAP0101 - Hypothetical protein
CAP0173 - Archaeal-type Fe-S oxidoreductase
CAP0174 - Membrane protein
CAP0040 - Xre family DNA-binding domain & TPR repeat protein
CAP0084 - Hypothetical protein
CAP0088 - abf
CAP0104 - arsB
CAP0105 - arsC
CAP0148 - phlC
CAP0025 - pdc
CAP0123 - Hypothetical protein
CAP0165 - adc
CAP0072 - Hypothetical protein
CAP0135 - Oxidoreductase
CAP0136 - AstB/chuR/nirj-related protein
CAP0137 - Similar to UDP-glucuronosyltransferases
CAP0133 - Antibiotic-resistance protein
CAP0134 - Hypothetical protein
CAP0138 - Distantly related to biotin carboxylase
CAP0139 - Possible D-alanyl carrier protein
CAP0053 - xynB
CAP0054 - Xylanase/chitin deacetylase family enzyme
CAP0150 - spoVD
CAP0047 - Sulphohydrolase/Glycosulfatase
CAP0085 - Hypothetical secreted protein
CAP0129 - Glycogen-binding subunit of S/T protein phosphatase I
CAP0112 - Hypothetical protein
CAP0086 - Permease
CAP0094 - tnpA
CAP0034 - Rhodanese-like domain
CAP0064 - alf
CAP0144 - Possible steroid binding protein
CAP0012 - pedB
CAP0091 - Predicted regulator of stationary gene expression
CAP0081 - TnpR resolvase C-terminal fragment
CAP0015 - GLPQ related phosphodiesterase
CAP0097 - lipG
CAP0102 - Membrane protein
CAP0163 - ctfA
CAP0164 - ctfB
CAP0162 - adhE1
CAP0009 - Response regulator
CAP0083 - SAM-dependent methyltransferase
CAP0095 - Recombinase XerD fragment
CAP0116 - Xylanase
CAP0156 - Hypothetical protein
CAP0010 - bglA
CAP0070 - HAD phosphatase superfamily protein
CAP0141 - mbhS
CAP0142 - mbhL
CAP0124 - Permease
CAP0087 - HTH transcriptional regulator
CAP0175 - repA
CAP0126 - Uncharacterized protein
CAP0006 - GntR family HTH transcriptional regulator
CAP0149 - Xre family DNA-binding domain & TRP repeat protein
CAP0026 - Hypothetical protein
CAP0100 - HTH transcriptional regulator
CAP0028 - HTH transcriptional regulator
CAP0131 - Permease
CAP0050 - pnbA
CAP0127 - HTH transcriptional regulator
CAP0055 - Conserved membrane protein
CAP0024 - HTH transcriptional regulator
CAP0154 - Membrane protein
CAP0151 - Integrin-like repeats domain fused to lysozyme
CAP0152 - Hypothetical protein
CAP0068 - ptnD
CAP0066 - ptnA
CAP0067 - manY/levF
CAP0069 - Uncharacterized protein
CAP0062 - Hypothetical protein
CAP0048 - Related to methyl-accepting chemotaxis protein
CAP0003 - Transglutaminase-like predicted protease domain
CAP0049 - Permease
CAP0002 - Transglutaminase-like predicted protease domain
CAP0051 - Oxidoreductase
CAP0117 - Possible beta-xylosidase diverged
4-fold lower than 1st timepoint
4-fold higher than 1st timpoint
Expression scale
1
50
100
Rank scale

Figure S7 - Expression profiles of genes on the pSOL1 megaplasmid of *C. acetobutylicum*

Genes with more than eight consecutively expressed timepoints were hierarchically clustered using the Pearson correlation. Because of the cutoff of eight timepoints, forty genes were not clustered. Arrows denote the onset of transitional phase. Gray squares indicate timepoints at which the intensity did not exceed the threshold value. (a) Expression values are presented as ratios compared to the first expressed timepoint, with genes having a higher expression shown as red and those with a lower expression as green. Saturated expression level: 4-fold difference (see scale). (b) Ranked expression intensity values for each gene. Ranks run from 100 to 1 with 100 being blue and 1 being white (see scale).

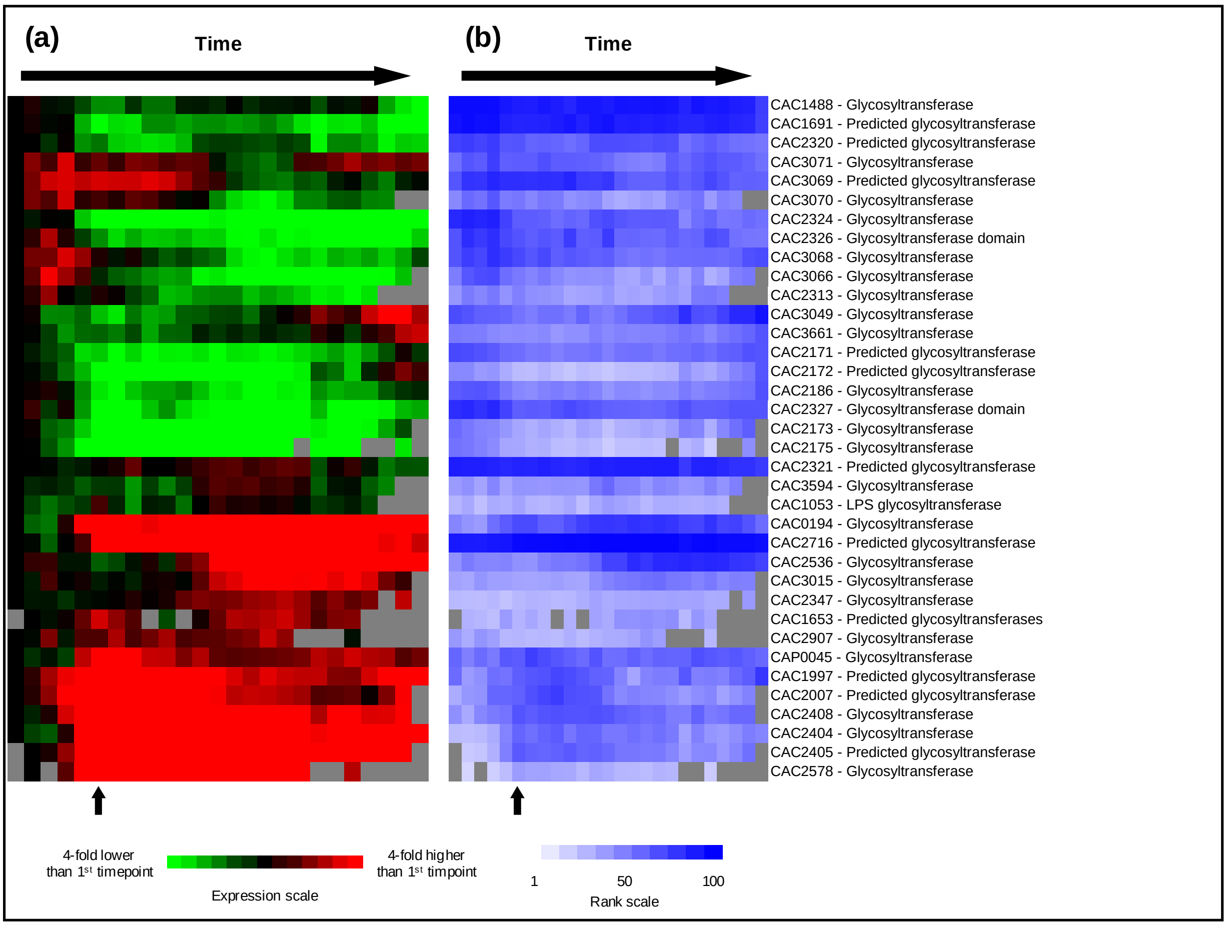

Figure S8 - Expression profiles of glycosyltransferases identified on the *C. acetobutylicum* genome

Genes with more than eight consecutively expressed timepoints were hierarchically clustered using the Pearson correlation. Because of the cutoff of eight timepoints, twenty-two genes were not clustered. Arrows denote the onset of transitional phase. Gray squares indicate timepoints at which the intensity did not exceed the threshold value. (a) Expression values are presented as ratios compared to the first expressed timepoint, with genes having a higher expression shown as red and those with a lower expression as green. Saturated expression level: 4-fold difference (see scale). (b) Ranked expression intensity values for each gene. Ranks run from 100 to 1 with 100 being blue and 1 being white (see scale).

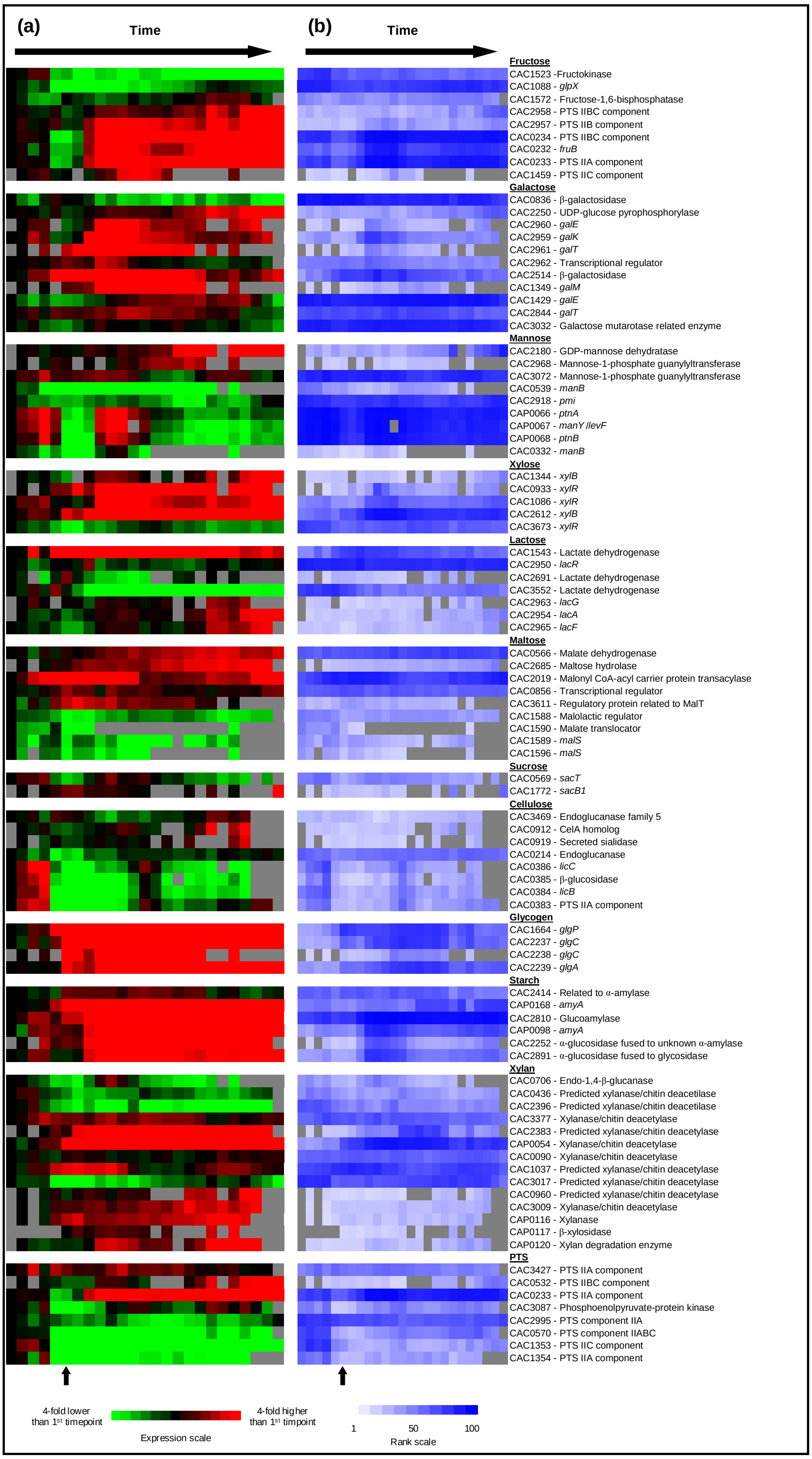
(a)
Time
(b)
Time
Fructose
CAC1523 -Fructokinase
CAC1088 - glpX
CAC1572 - Fructose-1,6-bisphosphatase
CAC2958 - PTS IIBC component
CAC2957 - PTS IIB component
CAC0234 - PTS IIBC component
CAC0232 - fruB
CAC0233 - PTS IIA component
CAC1459 - PTS IIC component
Galactose
CAC0836 - β-galactosidase
CAC2250 - UDP-glucose pyrophosphorylase
CAC2960 - galE
CAC2959 - galK
CAC2961 - galT
CAC2962 - Transcriptional regulator
CAC2514 - β-galactosidase
CAC1349 - galM
CAC1429 - galE
CAC2844 - galT
CAC3032 - Galactose mutarotase related enzyme
Mannose
CAC2180 - GDP-mannose dehydratase
CAC2968 - Mannose-1-phosphate guanylyltransferase
CAC3072 - Mannose-1-phosphate guanylyltransferase
CAC0539 - manB
CAC2918 - pmi
CAP0066 - ptnA
CAP0067 - manY /levF
CAP0068 - ptnB
CAC0332 - manB
Xylose
CAC1344 - xylB
CAC0933 - xylR
CAC1086 - xylR
CAC2612 - xylB
CAC3673 - xylR
Lactose
CAC1543 - Lactate dehydrogenase
CAC2950 - lacR
CAC2691 - Lactate dehydrogenase
CAC3552 - Lactate dehydrogenase
CAC2963 - lacG
CAC2954 - lacA
CAC2965 - lacF
Maltose
CAC0566 - Malate dehydrogenase
CAC2685 - Maltose hydrolase
CAC2019 - Malonyl CoA-acyl carrier protein transacylase
CAC0856 - Transcriptional regulator
CAC3611 - Regulatory protein related to MalT
CAC1588 - Malolactic regulator
CAC1590 - Malate translocator
CAC1589 - malS
CAC1596 - malS
Sucrose
CAC0569 - sacT
CAC1772 - sacB1
Cellulose
CAC3469 - Endoglucanase family 5
CAC0912 - CelA homolog
CAC0919 - Secreted sialidase
CAC0214 - Endoglucanase
CAC0386 - licC
CAC0385 - β-glucosidase
CAC0384 - licB
CAC0383 - PTS IIA component
Glycogen
CAC1664 - glgP
CAC2237 - glgC
CAC2238 - glgC
CAC2239 - glgA
Starch
CAC2414 - Related to α-amylase
CAP0168 - amyA
CAC2810 - Glucoamylase
CAP0098 - amyA
CAC2252 - α-glucosidase fused to unknown α-amylase
CAC2891 - α-glucosidase fused to glycosidase
Xylan
CAC0706 - Endo-1,4-β-glucanase
CAC0436 - Predicted xylanase/chitin deacetilase
CAC2396 - Predicted xylanase/chitin deacetilase
CAC3377 - Xylanase/chitin deacetylase
CAC2383 - Predicted xylanase/chitin deacetylase
CAP0054 - Xylanase/chitin deacetylase
CAC0090 - Xylanase/chitin deacetylase
CAC1037 - Predicted xylanase/chitin deacetylase
CAC3017 - Predicted xylanase/chitin deacetylase
CAC0960 - Predicted xylanase/chitin deacetylase
CAC3009 - Xylanase/chitin deacetylase
CAP0116 - Xylanase
CAP0117 - β-xylosidase
CAP0120 - Xylan degradation enzyme
PTS
CAC3427 - PTS IIA component
CAC0532 - PTS IIBC component
CAC0233 - PTS IIA component
CAC3087 - Phosphoenolpyruvate-protein kinase
CAC2995 - PTS component IIA
CAC0570 - PTS component IIABC
CAC1353 - PTS IIC component
CAC1354 - PTS IIA component
4-fold lower than 1st timepoint
4-fold higher than 1st timpoint
Expression scale
1
50
100
Rank scale

Figure S9 - Expression profiles of genes related to various carbohydrate pathways and phosphotransferase system (PTS) genes

Arrows denote the onset of transitional phase. Gray squares indicate timepoints at which the intensity did not exceed the threshold value. (a) Expression values are presented as ratios compared to the first expressed timepoint, with genes having a higher expression shown as red and those with a lower expression as green. Saturated expression level: 4-fold difference (see scale). (b) Ranked expression intensity values for each gene. Ranks run from 100 to 1 with 100 being blue and 1 being white (see scale).

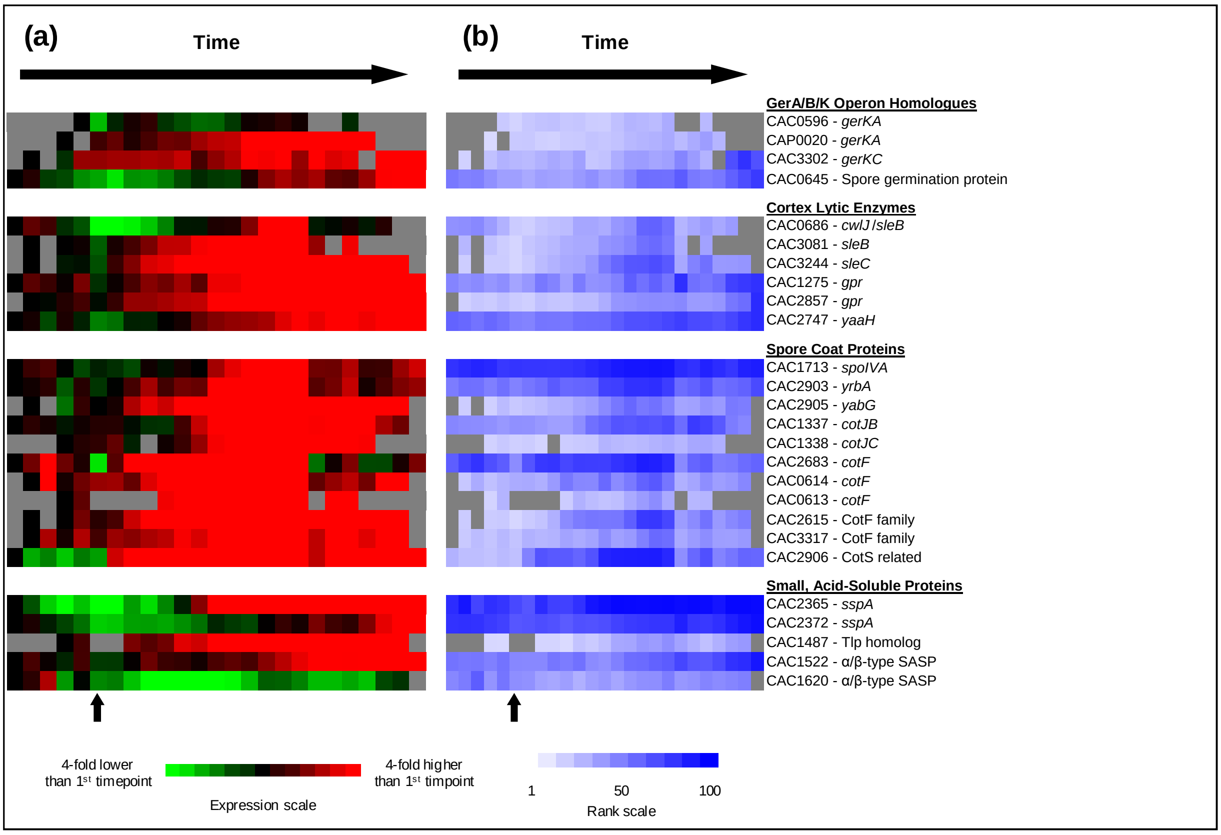

Figure S10 - Expression profiles of genes associated with germination, cortex lytic enzymes, spore coat proteins, and small, acid-soluble proteins identified on the *C. acetobutylicum* genome

Arrows denote the onset of transitional phase. Gray squares indicate timepoints at which the intensity did not exceed the threshold value. (a) Expression values are presented as ratios compared to the first expressed timepoint, with genes having a higher expression shown as red and those with a lower expression as green. Saturated expression level: 4-fold difference (see scale). (b) Ranked expression intensity values for each gene. Ranks run from 100 to 1 with 100 being blue and 1 being white (see scale).

Germination receptors, spore cortex lysis enzymes, and proteins of the spore coat and core

Though the regulation of sporulation has been examined in *C. acetobutylicum* [1], genes related to spore formation and function have not been as thoroughly investigated. Here, based on annotation and homology to known *B. subtilis* and other clostridia genes, we examined the expression profiles of genes relating to germination, cortex lytic enzymes, spore coat proteins, and small, acid-soluble proteins. Because dormant spores cannot synthesize macromolecules, the development of germination machinery is an integral part of sporulation [68]. Germination receptor proteins initiate germination upon binding of an extracellular signal (e.g., the binding of L-alanine by the GerA receptor in *B. subtilis*). In *C. acetobutylicum*, fourteen homologous *gerA/B/K* genes exist; four exist on pSOL1 [22]. Of those, only four are above the expression threshold. Three genes (CAC0645, CAC3302, and CAP0020) were upregulated late in the timecourse, while the fourth gene, CAC0496, was expressed at relatively constant levels between hours 10 to 36 (Figure S9). These results differ significantly from the transcriptional analysis of the homologous tricistronic operons in *B. subtilis (gerA*, *gerB*, *gerK*) [69]. Additionally, because none of the expressed genes are homologous to *gerAB*, it is unlikely that the typical GerAA/GerAB/GerAC receptor complex [68] can be formed in *C. acetobutylicum*. This suggests that either germinant receptors do not play a significant role in initiating germination in *C. acetobutylicum* or that the receptors have a different structure or are coded by unknown genes. *C. acetobutylicum*, unlike bacilli, does not sporulate as a result of nutrient deprivation [70], and so it is conceivable that its germination is not stimulated by nutrient availability either.

Core lytic enzymes (CLEs) selectively hydrolyze muramic-δ-lactam residues found only in cortex proteoglycans [71]. CLE activity begins shortly after germination signals are received and enables proper germination and outgrowth. The primary CLEs identified thus far in bacilli are

SleB and SleL, a lytic transglycosylase and N-acteylglucosaminidase, respectively [71]. Bacilli also possess CwlJ, an enzyme homologous to SleB that plays a somewhat redundant role to SleB, and YaaH, which displays high homology with SleL from *B. cereus* [71]. In bacilli, *cwlJ* and *selL* are regulated by σ^E [72,73] and *sleB* is regulated by σ^G [74]. The CLE system identified in *C. perfringens* consists of SleC and SleM [71]. Homologues of CwlJ, SleB, YaaH, and SleC have been identified in *C. acetobutylicum*. In addition to the CLEs, both bacilli and clostridia possess germination proteases (GPR) which degrade SASP-substrates to generate a source of amino acids [75]. Most of the genes were initially upregulated during the onset of stationary phase and increased in expression throughout the timecourse. *yaaH* had a delayed expression compared to the other genes but was still highly upregulated by the end of the timecourse. The only exception was CAC0686 (*cwlJ*/*sleB*) which was not expressed till hour 30 and then sharply dropped off after hour 36.

Spore coat proteins protect the cell from toxic compounds and may also play a role in germination [76]. Spore coat proteins identified in *C. acetobutylicum* are: CotJB, CotJC, CotF, and CotS. Several proteins involved in the assembly of the spore coat were also identified: SpoIVA, a protein responsible for spore-coat assembly in *B. subtilis* [77], YrbA, an assembly protein, and YabG, an ortholog of the *B. subtilis* protease which processes several spore coat proteins [78]. In *B. subtilis*, *spoIVA*, *yrbA*, and the *cotJ* operon are under the control of σ^E, while *cotF* and *yabG* is under the control of σ^K, and *cotS* is under the control of both σ^K and GerE [79]. Many genes (CAC2905, CAC2683, CAC0614, CAC0613, CAC2615, CAC3317, and CAC2906) were all upregulated at the onset of stationary phase, consistent with many other sporulation-related genes, while the remaining genes (CAC1713, CAC2903, CAC1337, and CAC1338) were

upregulated about 8 hours later. The genes remained upregulated until the end of the timecourse, except for CAC2683, which dropped off at hour 42, and CAC1713, CAC2903, CAC0614, and CAC0613, which decreased in expression but remained upregulated.

SASPs non-specifically bind genomic DNA in dormant spores, providing protection against damaging agents and are divided into three classes: α, β, and γ. In bacilli, while deletion of α/β-type SASPs increase spore sensitivity to damaging agents, simultaneous deletion of the two primary α/β-type SASPs, SspA and SspB, affects several aspects of the sporulation and germination transcriptional programs, implying that SASPs may also affect gene expression at a global level during both processes [80]. In *C. acetobutylicum*, four α/β-type SASPs are annotated: two are homologs of the σ^G-dependent SspA found in *B. subtilis* [80], while two other α/β-type SASPs (CAC1522, CAC1620) possess no identifiable *B. subtilis* homologs. Also found on the genome is a homolog of Tlp (a σ^F/σ^G-dependent SASP) [80]. One of the *sspA* homologs (CAC2365) began to increase in expression around hour 22 and stayed highly upregulated throughout the timecourse, while the other homolog (CAC2372) did not increase in expression until hour 36. CAC1487, the *tlp* homolog, was upregulated shortly after stationary phase onset and remained highly expressed. Of the two uncharacterized SASP genes, only CAC1522 was upregulated, at hour 26, while CAC1620 actually decreased in expression throughout the experiment.

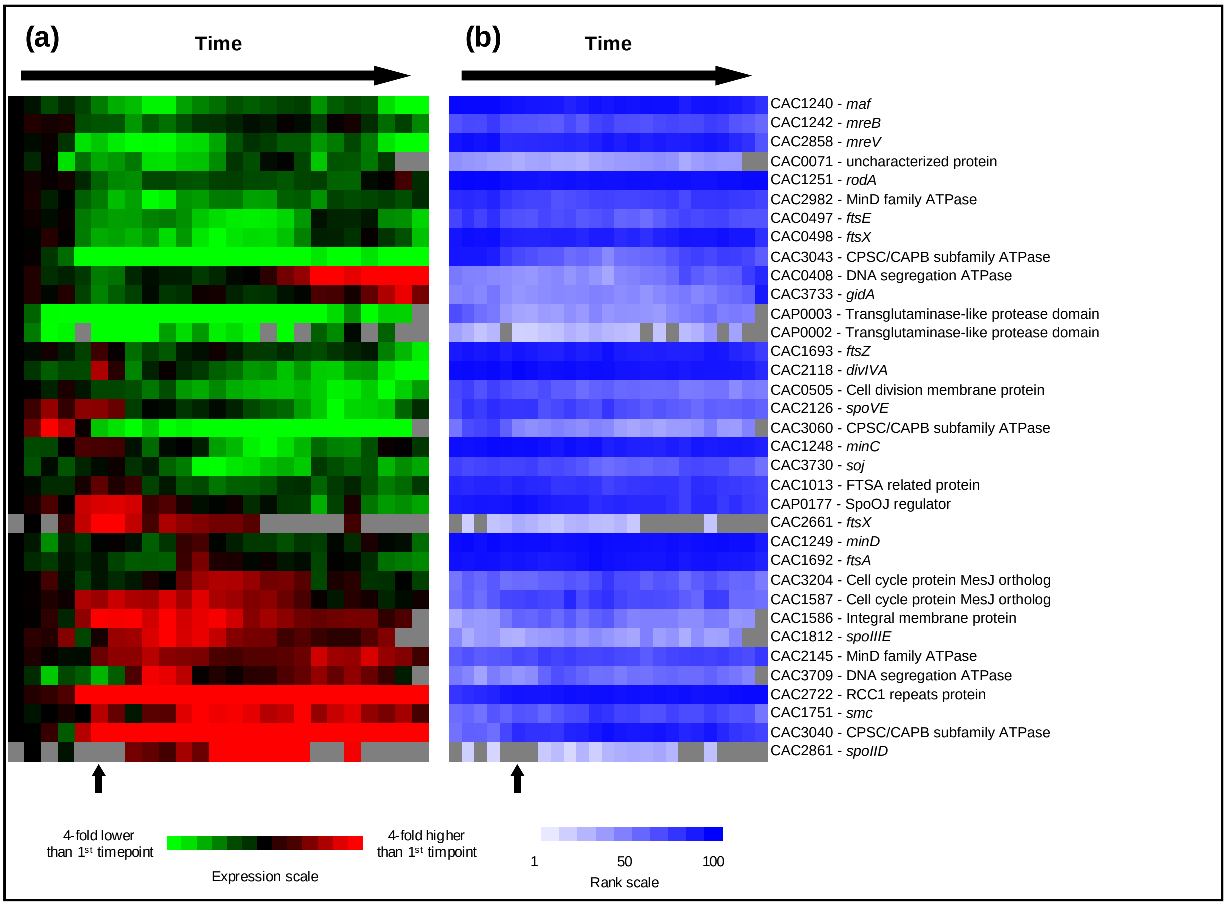

Figure S11 - Expression profiles of genes related to cell division and chromosome partitioning (COG class D)

Genes with more than eight consecutively expressed timepoints were hierarchically clustered using the Pearson correlation. Because of the cutoff of eight timepoints, three genes within this COG class were not clustered. Arrows denote the onset of transitional phase. Gray squares indicate timepoints at which the intensity did not exceed the threshold value. (a) Expression values are presented as ratios compared to the first expressed timepoint, with genes having a higher expression shown as red and those with a lower expression as green. Saturated expression level: 4-fold difference (see scale). (b) Ranked expression intensity values for each gene. Ranks run from 100 to 1 with 100 being blue and 1 being white (see scale).

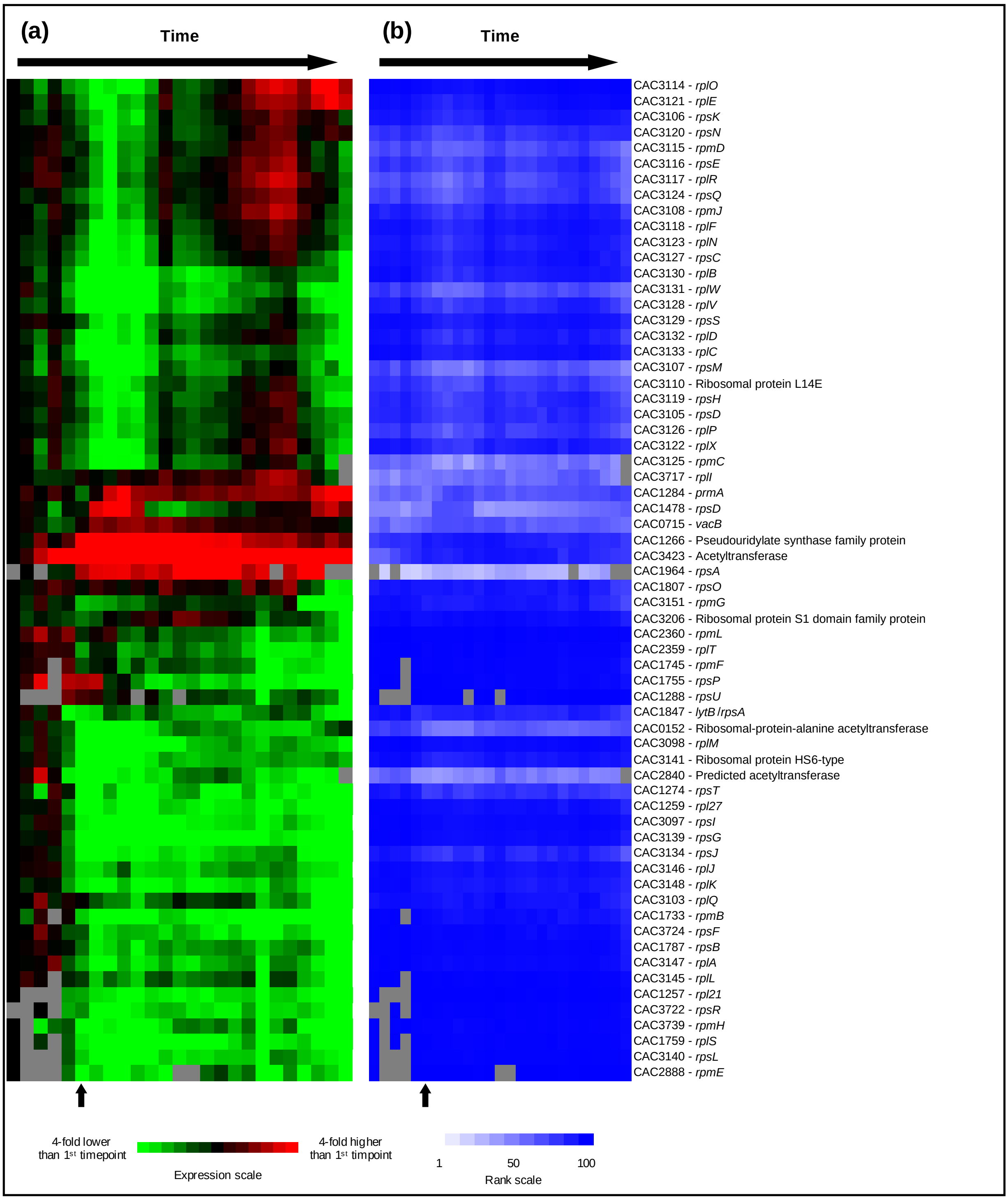

Figure S12 - Expression profiles of ribosomal proteins identified on the *C. acetobutylicum* chromosome

Genes with more than eight consecutively expressed timepoints were hierarchically clustered using the Pearson correlation. Because of the cutoff of eight timepoints, one gene was not clustered. Arrows denote the onset of transitional phase. Gray squares indicate timepoints at which the intensity did not exceed the threshold value. (a) Expression values are presented as ratios compared to the first expressed timepoint, with genes having a higher expression shown as red and those with a lower expression as green. Saturated expression level: 4-fold difference (see scale). (b) Ranked expression intensity values for each gene. Ranks run from 100 to 1 with 100 being blue and 1 being white (see scale).
