# Supplementary material for: The transcriptional program underlying the physiology of clostridial sporulation
Source: Genome Biol. 2008 Jul 16;9(7):R114. doi: 10.1186/gb-2008-9-7-r114 (PMC2530871; doi:10.1186/gb-2008-9-7-r114)
Supplement: Additional data file 4 — Includes a brief discussion on how the genes used to construct the deduced activity plots were chosen. [file gb-2008-9-7-r114-S4.pdf]

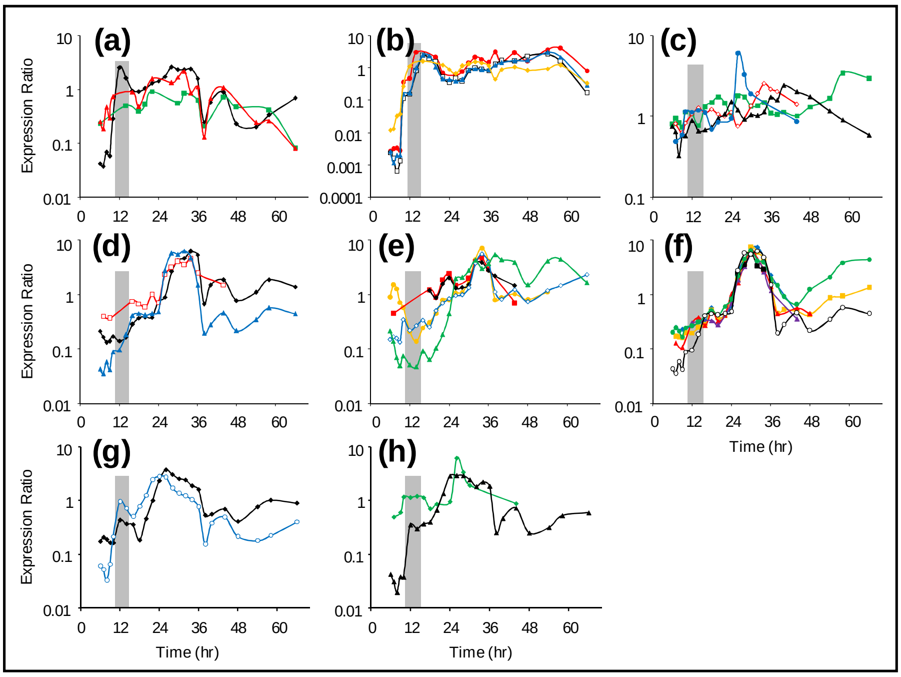

Figure S13 - Expression profiles of genes which were standardized

Expression values are ratios against the reference RNA pool. Gray bar indicates the onset of transitional phase. (a) Putative genes under the control of σ^{H}: *glgP* (CAC1664, ♦), *spoVS* (CAC1817, ■), and *spoVG* (CAC3223, ▲). (b) Putative genes under the control of Spo0A~P: *adhE1* (CAP0162, ●), *ctfA* (CAP0163, □), *ctfB* (CAP0164, ▲), and *adc* (CAP0165, ♦). (c) Putative genes under the control of σ^{F}: *lonB* (CAC2638, ◊), *lonB* (CAC3716, ▲), *csfB* (CAC0296, ■), and *spoIIR* (CAC2898, ●). (d) Putative genes under the control of σ^{E}: *spoVR* (CAC0581, ♦), *spoIID* (CAC2861, □), and *spoIIIAH* (CAC2086, ▲). (e) Putative genes under the control of σ^{G}: *spoVAD* (CAC2304, ♦), *spoVAC* (CAC2305, ■), *sspA* (CAC2365, ▲), *sleB* (CAC0686, ●), and *spoVT* (CAC3214, ◊). (f) Genes within the predicted *spoIIIA* operon: *spoIIIAA* (CAC2093, ♦), *spoIIIAB* (CAC2092, ■), *spoIIIAC* (CAC2091, ▲), *spoIIIAD* (CAC2090, ▲), *spoIIIAF* (CAC2088, ■), *spoIIIAG* (CAC2087, ●), and *spoIIIAH* (CAC2086, ○).

(g) *sigF* (○) and *spoIIE* (CAC3205, ♦), the phosphatase responsible for σ^F activation. (h) *sigE* (▲) and *spoIIR* (CAC2898, ♦), a protein needed for proper pro-σ^E processing.

Gene selection for deduced activity plots

In order to construct the deduced activity plots (Figure 4), known or robustly identifiable canonical genes were selected for each sporulation-related factor. For σ^H activity, genes identified to be within the σ^H regulon in *B. subtilis* [81], were solely under the control of σ^H, and had a predicted σ^H binding motif in *C. acetobutylicum* [37] were chosen. Genes which met this criteria were: *glgP* (CAC1664), *spoVS* (CAC1817), and *spoVG* (CAC3223). All three of these genes displayed similar expression (Figure S13a). *adhE1* (CAP0162), *ctfA* (CAP0163), *ctfB* (CAP0164), and *adc* (CAP0165) were used for Spo0A activity because of their known regulation by Spo0A~P (Figure S13b) [29]. Based on the σ^F regulon in *B. subtilis* [82], a number of homologs were found in *C. acetobutylicum*, but many of these were also regulated by σ^G [82]. Also, because of this overlap, the binding motifs of σ^F and σ^G are very similar [82], and only a σ^F/σ^G promoter is predicted in *C. acetobutylicum* and not a separate σ^F and σ^G [37]. The genes identified to be under the sole regulation of σ^F and had a σ^F/σ^G binding motifs were: *csfB* (CAC0296), *spoIIR* (CAC2898), *lonB* (CAC2638), and *lonB* (CAC3716). Of these, only *spoIIR* had a single sharp peak (Figure S13c), but the general trend in all of them in similar. As with σ^F, a number of homologs for σ^E-controlled genes [83] were easily found, but only a handful had predicted σ^E binding motifs: *spoVR* (CAC0581), *spoIID* (CAC2861), *spoIIIAH* (CAC2086), and *spoIIM* (CAC2068). However, unlike the other three genes (Figure S12d), *spoIIM* expression peaked early during late exponential phase and did not peak again (data not shown), so it was not used in the activity profile. Homologs of genes solely under the control of σ^G [82] with a σ^F/σ^G

binding motif were: *spoVAD* (CAC2304), *spoVAC* (CAC2305), *sspA* (CAC2365), *sleB* (CAC0686), *spoVT* (CAC3214), and *gerKA* (CAC0596). All displayed similar peaks during mid-stationary phase (Figure S12e), except *gerKA* which peaked early during late exponential phase and was not used to construct the activity profile (data not shown).

After selecting the genes, both the regulon genes' and the transcription factor's expression profiles were standardized to adjust for differences in relative expression levels [7]. To do this, the expression ratio for each gene at each timepoint was divided by the square root of the sum of the squares of that gene's expression ratios, so that the sum of the squares of the ratios for each gene would be one. The regulon genes' ratios were then averaged together to produce the activity plots.
