# Supplementary material for: The transcriptional program underlying the physiology of clostridial sporulation
Source: Genome Biol. 2008 Jul 16;9(7):R114. doi: 10.1186/gb-2008-9-7-r114 (PMC2530871; doi:10.1186/gb-2008-9-7-r114)
Supplement: Additional data file 5 — Differential expression and intensity of all annotated histidine kinases and response regulators. [file gb-2008-9-7-r114-S5.pdf]

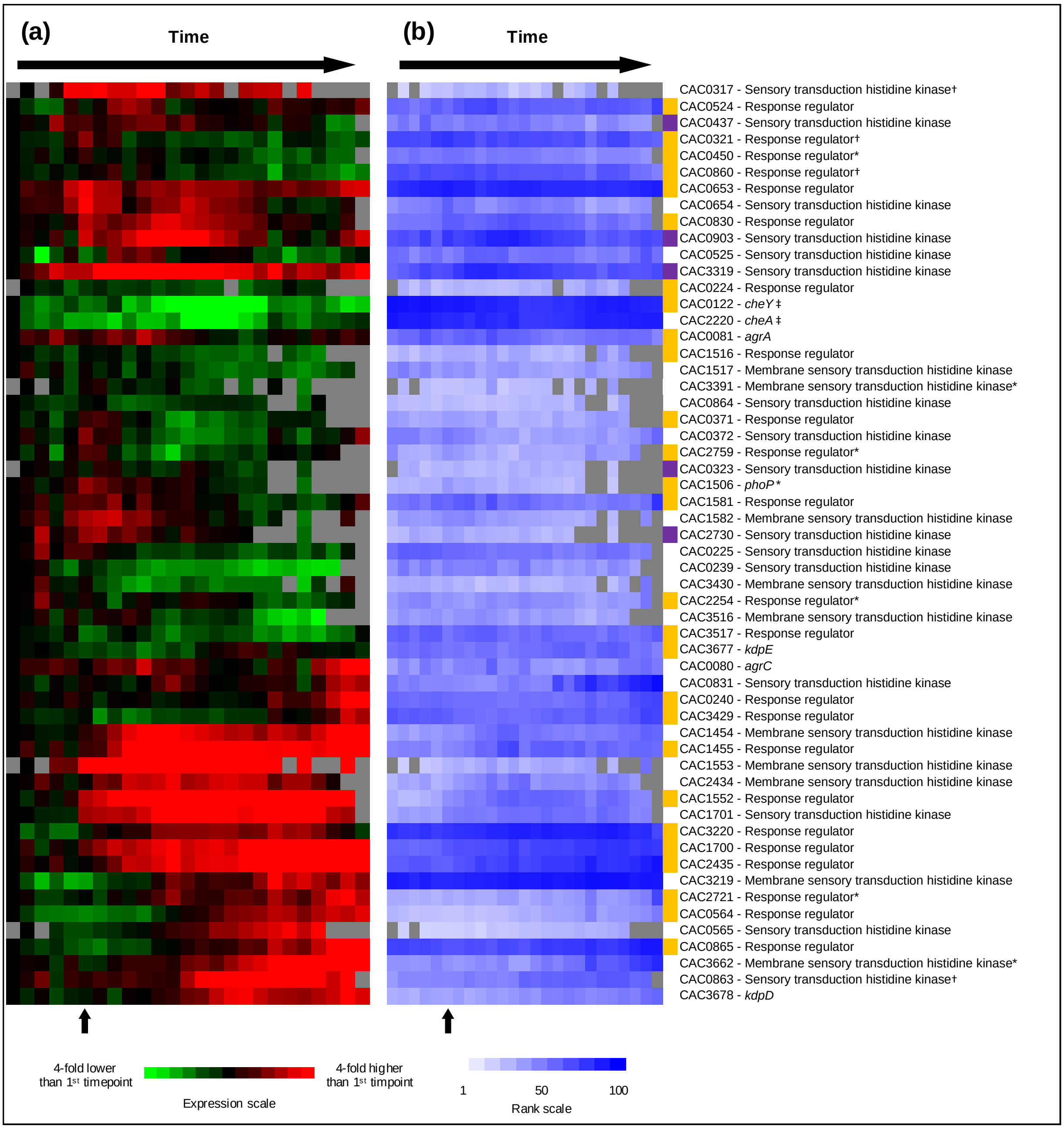

Figure S14 - Expression profiles of sensory histidine kinases and their associated response regulators identified on the *C. acetobutylicum* chromosome

Orphan kinases (kinases without an adjacent response regulator) are denoted by purple boxes, and response regulators are denoted by orange boxes. Asterisks (*) denote genes whose associated histidine kinase or response regulator was eliminated because of the eight

consecutively expressed timepoints criteria. Genes which met this cutoff were hierarchically clustered using the Pearson correlation. Because of the cutoff of eight timepoints, eleven genes were not clustered. Crosses (†) denote kinases and response regulators on the same predicted operon but not adjacent to each other. Double crosses (‡) denote genes known to interact, but not located adjacent to each other or on the same operon. Arrows denote the onset of transitional phase. Gray squares indicate timepoints at which the intensity did not exceed the threshold value. (a) Expression values are presented as ratios compared to the first expressed timepoint, with genes having a higher expression shown as red and those with a lower expression as green. Saturated expression level: 4-fold difference (see scale). (b) Ranked expression intensity values for each gene. Ranks run from 100 to 1 with 100 being blue and 1 being white (see scale).
