# Supplementary material for: The transcriptional program underlying the physiology of clostridial sporulation
Source: Genome Biol. 2008 Jul 16;9(7):R114. doi: 10.1186/gb-2008-9-7-r114 (PMC2530871; doi:10.1186/gb-2008-9-7-r114)
Supplement: Additional data file 6 — Phylogenetic tree resulting from the alignment of the σ70-related and unannotated sigma factors from ten bacterial species. [file gb-2008-9-7-r114-S6.pdf]

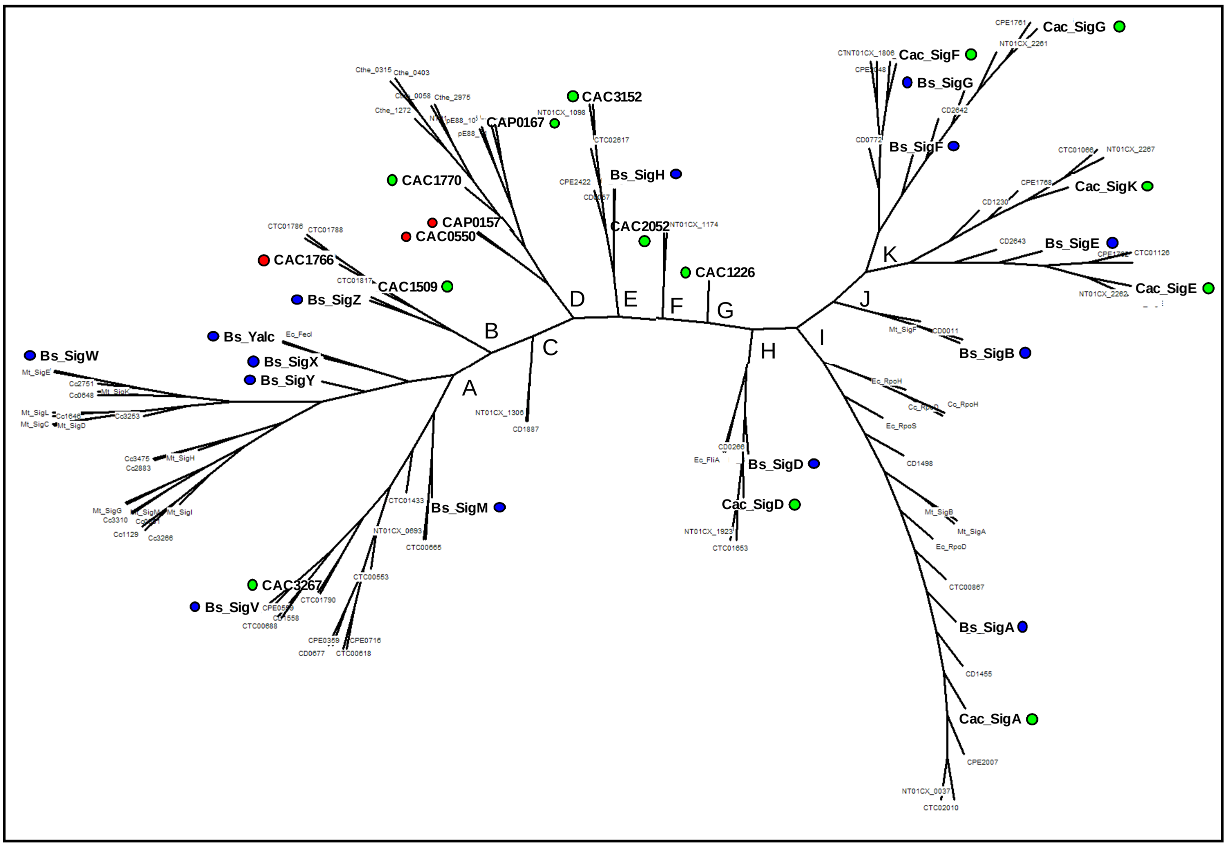

Figure S15 - Phylogenetic tree resulting from the alignment of the σ^{70}-family and unannotated sigma factors from 10 bacterial species

σ^{70}-family and unannotated sigma factors from *C. acetobutylicum* (CAC and CAP), *E. coli* (EC), *B. subtilis* (Bs), *C. crecentus* (Cc), *M. tuberculosis* (Mt), *C. thermocellum* (Cthe), *C. tetani* E88 (CTC and pE88), *C. perfringens* strain 13 (CPE), *C. difficile* (CD), and *C. novyi* NT (NT01CX) were filtered for a Region 2, because Region 2 of σ^{70} is the most conserved region of the entire protein, and were then aligned based on the entire sequence. It should be noted that although CAC1766, CAP0157, and CAC0550 do not contain a Region 2, they were also included in the alignment for completeness. These three sigma factors are indicated by a red dot before their names, while the remaining *C. acetobutylicum* sigma factors and the *B. subtilis* sigma factors are indicated by a green dot and a blue dot, respectively. The branches can be classified as: extracytoplasmic function (ECF) (A), unknown (possible ECF family) (B), unknown (possible

ECF family) (C), unknown (D), *sigH* (E), unknown (possible ECF family) (F), *sigA* annotated (G), *sigD* (H), *sigA* (I), *sigB* (J), and sporulation (*sigF*, *sigE*, and *sigG*) (K).
