# Supplementary material for: The transcriptional program underlying the physiology of clostridial sporulation
Source: Genome Biol. 2008 Jul 16;9(7):R114. doi: 10.1186/gb-2008-9-7-r114 (PMC2530871; doi:10.1186/gb-2008-9-7-r114)
Supplement: Additional data file 8 — TEM images of the plasmid control strain, asCAP0167, and asCAC1766. [file gb-2008-9-7-r114-S8.pdf]

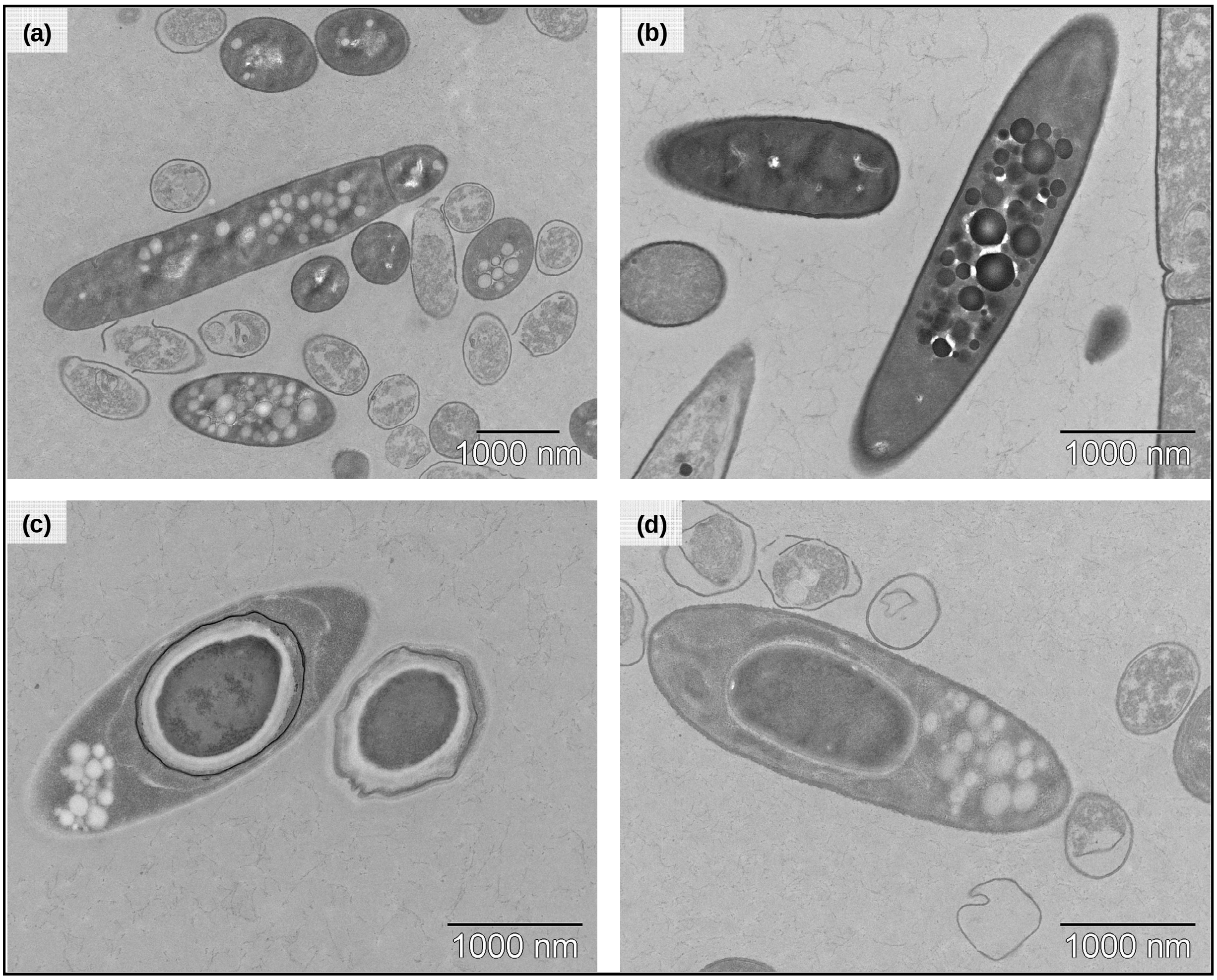

Figure S16 - Additional transmission electron microscopy (TEM) images from the plasmid control strain pSOS95del

TEM images from a differentiating pSOS95del culture. (a) An elongated clostridial form, with typical electron translucent granulose, probably undergoing asymmetric division. Also visible is a shorter clostridial form with electron translucent granulose. (b) Elongated clostridial form with granulose accumulation, but the granulose is electron dense instead of electron translucent. (c) Typical endospore form and a free mature spore. Electron translucent granulose is still visible within the endospore form. (d) Typical endospore form with electron translucent granulose.

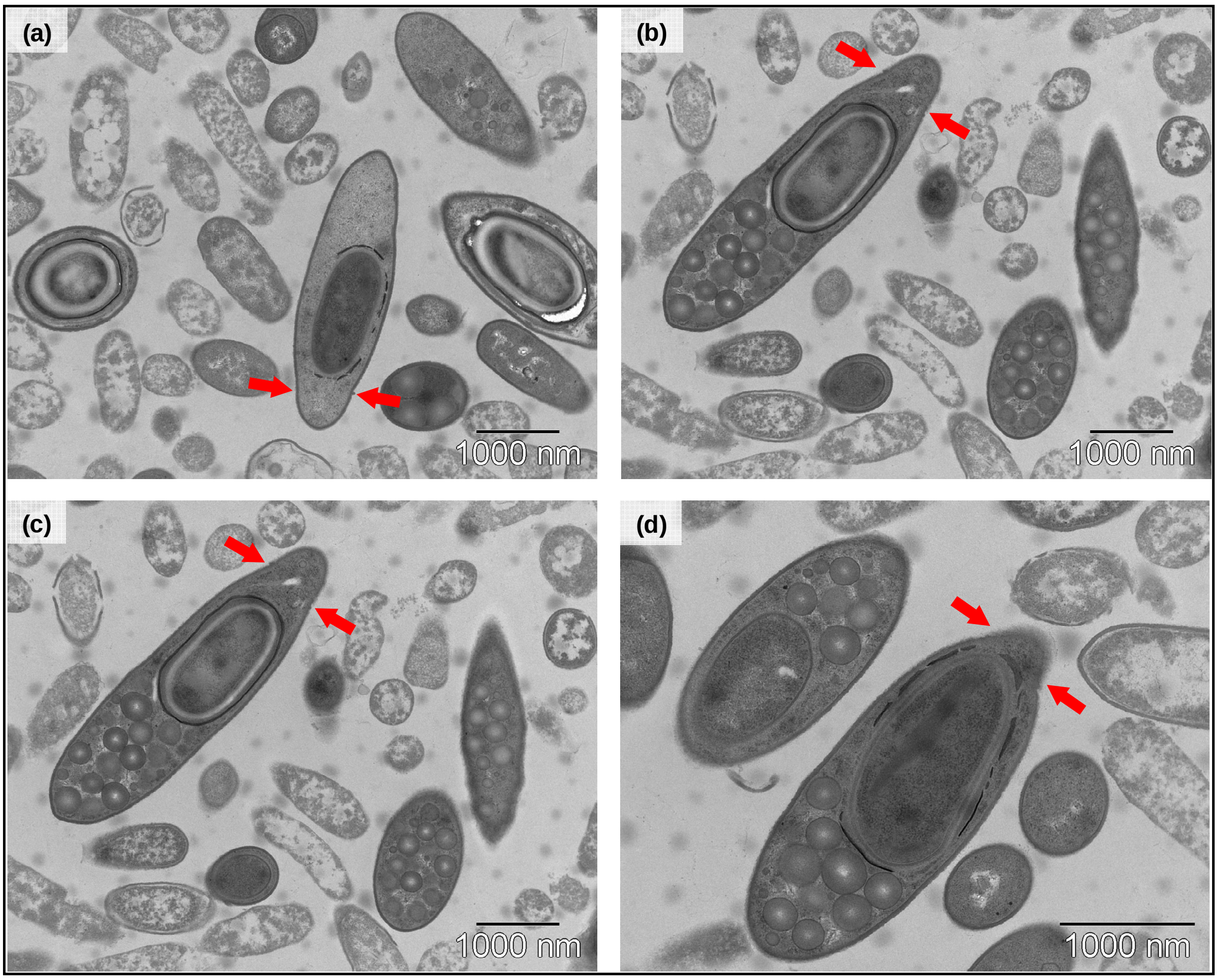

Figure S17 - Additional transmission electron microscopy (TEM) images from the antisense strain against CAP0167 (asCAP0167)

TEM images of the asCAP0167 strain. Red arrows indicate pinched ends not seen in the plasmid control samples. Noticeable also are the electron dense granules with a well-defined perimeter, in contrast to the electron translucent granules without a defined perimeter in the control strain (Figure S16).

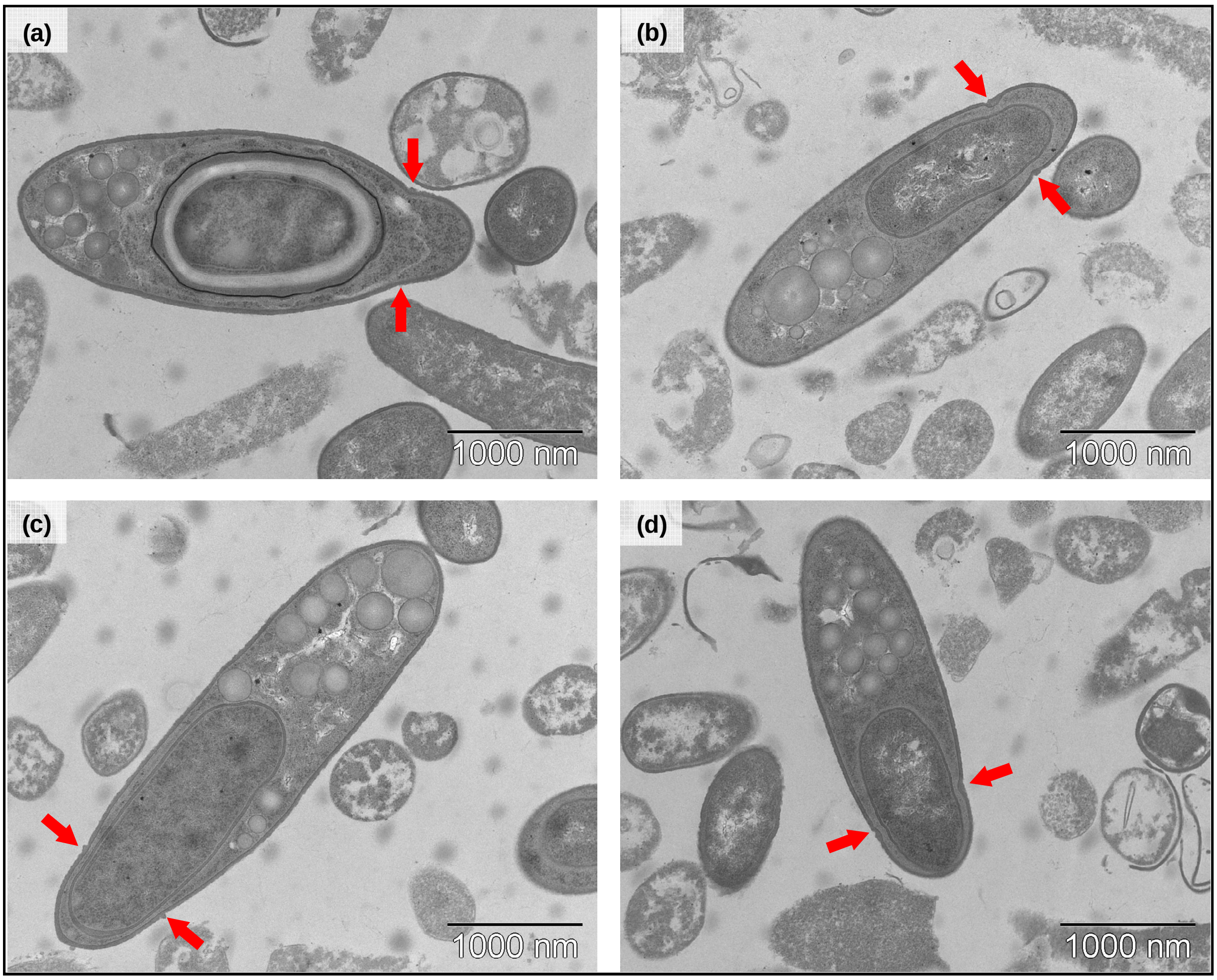

Figure S18 - Additional transmission electron microscopy (TEM) images from the antisense strain against CAC1766 (asCAC1766)

TEM images of the asCAC1766 strain. Red arrows indicate pinched ends not seen in the plasmid control samples. Noticeable also are the large, electron dense granules with a well-defined perimeter, in contrast to the electron translucent and smaller granules without a defined perimeter in the control strain (Figure S16).
